# Recurring urothelial carcinomas show genomic rearrangements incompatible with a direct relationship

**DOI:** 10.1038/s41598-020-75854-4

**Published:** 2020-11-11

**Authors:** Nour-Al-Dain Marzouka, David Lindgren, Pontus Eriksson, Gottfrid Sjödahl, Carina Bernardo, Fredrik Liedberg, Håkan Axelson, Mattias Höglund

**Affiliations:** 1 Division of Oncology, Department of Clinical Sciences, Lund University, Lund, Sweden; 2 Division of Translational Cancer Research, Department of Laboratory Medicine, Lund University, Lund, Sweden; 3 Division of Urological Research, Department of Translational Medicine, Malmö University Hospital, Malmö, Sweden; 4 Department of Urology, Skåne University Hospital, Malmö, Sweden

**Keywords:** Cancer genomics, Oncogenes, Bladder cancer

## Abstract

We used the fact that patients with non-muscle invasive bladder tumors show local recurrences and multiple tumors to study re-initiation of tumor growth from the same urothelium. By extensive genomic analyses we show that tumors from the same patient are clonal. We show that gross genomic chromosomal aberrations may be detected in one tumor, only to be undetected in a recurrent tumor. By analyses of incompatible changes i.e., genomic alterations that cannot be reversed, we show that almost all tumors from a single patient may show such changes, thus the tumors cannot have originated from each other. As recurring tumors share both genomic alterations and driver gene mutations, these must have been present in the urothelium in periods with no tumor growth. We present a model that includes a growing and evolving field of urothelial cells that occasionally, and locally, produce bursts of cellular growth leading to overt tumors.

## Introduction

There are two forms of urothelial carcinoma (UC), non-muscle invasive (NMI) and muscle invasive (MI), a definition of both genetic and clinical importance. Studies of muscle invasive UC have been dominating, as these patients show a worse survival and often are subjected to cystectomy. Patients with NMI UC frequently show local recurrences at distant sites within the bladder wall, often with long periods without tumors^1^. The question has been raised if the frequent recurrences are caused by de novo tumors or if they derive from previous overt tumors either by seeding or by migration^2^. However, an alternative hypothesis has been proposed suggesting that the urothelial carcinoma originates from a field cancerization phenomenon in which a single clonal area, sometimes referred to as a cancerized field, expands at the cost of neighboring clonal areas^2,3^. This phenomenon could be related to the finding that many types of normal surface epithelia are composed of clonal areas that replenish the tissue during normal homeostasis^4,5^.

Early studies from the Czerniak group^6^ used genetic and genomic mapping of cystectomized bladders to convincingly show that genomic alterations and gene mutations present in the overt tumors may already exist in the pathologically/morphologically normal urothelium surrounding the tumors^7^. The mapping of the normal mucosa from patients with UC revealed that the fields were highly heterogenous, indicating an ongoing genomic evolution and genetic divergence. This finding has recently been shown to be true also for other tumor types^8^. Similar detailed molecular analyses of pathologically normal sections of the bladder from patients with NMI tumors remains scarce, as these patients rarely are subjected to cystectomy. However, Thomsen et al.^9^ and Strandgaard et al.^10^ approached the problem by multiple sampling of tumor free regions of the urothelium from bladders with non-muscle invasive tumors. These studies revealed, although with less substantial data, the presence of genetic and genomic alterations in the pathologically normal urothelium of patients also with NMI tumors. Taken together, these studies provide important understandings of the genetic heterogeneity of the adjoining normal urothelium in patients with UC and have revealed insights into the processes behind tumor initiation and spread. We have taken a different approach and instead genetically analyzed recurring tumors from the same patient, allowing us to define the relationship between spatially and temporally distinct recurring tumors from the same patient. We focus on genomic alterations occurring in both metachronous and synchronous tumors from the same patient. We show that all meta- and synchronous tumors share many complex chromosomal aberrations, seen in recurrences during several years, while other complex aberrations may be absent in recurring tumors. Importantly, the majority of tumors from the same patient show aberrations not compatible with an origin directly from a previous or a neighboring tumor. Hence, our data suggest an extensive ongoing genomic evolution taking place in the tumor free urothelium resulting in locally recurring tumors.

## Results

**Figure 1:**
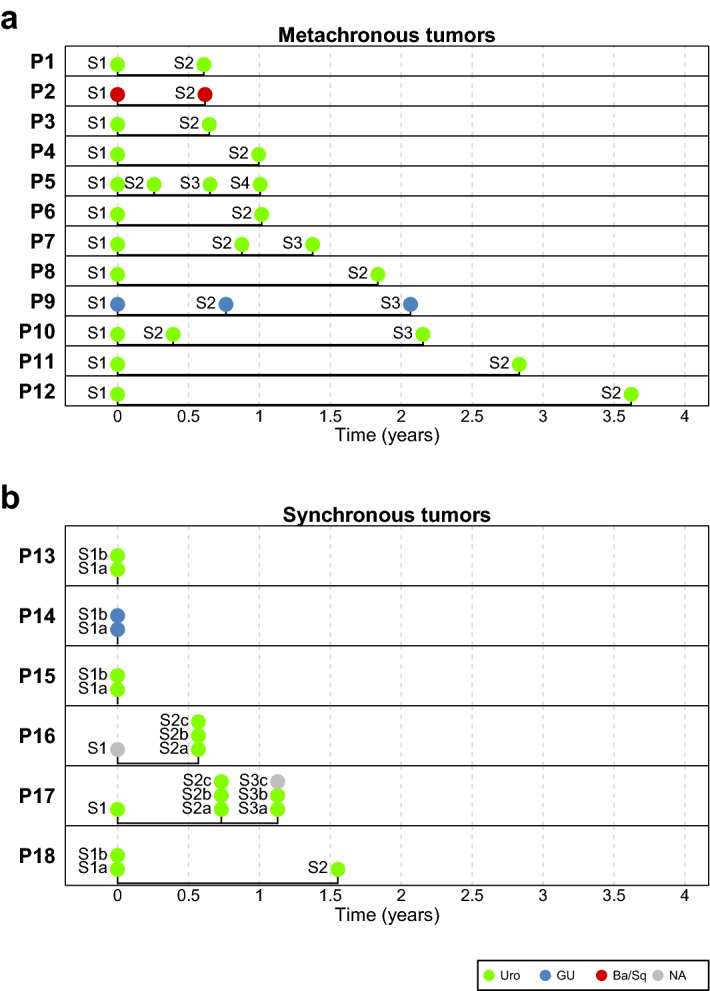
Timeline of recurring tumors. (**a**) Patients with metachronous tumors only. (**b**) Patients with synchronous tumors. UC numbers represent the patient IDs. Tumors are colored coded according to molecular subtype. Urothelial-like (Uro), green; Genomically Unstable (GU), blue; Basal/Squamous-like (Ba/Sq), red; not determined, gray. Figure was created using R v3.6 (https://www.r-project.org/).

The present cohort comprise a total of 48 metachronous and synchronous tumors from 18 patients. For the 12 patients with metachronous tumors, the time between the first and the last tumor ranged from four months to 3.5 years (Fig. 1a). Six patients with synchronous tumors were analyzed, one patient presented with synchronous tumors at two time points (Fig. 1b). Based on gene expression data, tumors were classified according to Lund Taxonomy into Urothelial-like (Uro), Genomically Unstable (GU), Basal/Squamous-like (Ba/Sq), Small cell/Neuroendocrine-like (Sc/Ne), or Mesenchymal-like (Mes-like) (Fig. 1). To investigate possible genomic relationships and heterogeneity among metachronous and synchronous tumors, DNA were isolated and subjected to DNA copy number analyses. Based on these analyses we determined ploidy levels, allelic imbalances, genomic amplifications, and chromosomal breakpoints. For 27 of the tumors the mutational status was assessed by exome sequencing using a custom panel of 1697 cancer-relevant genes (Supplementary File 1). A detailed case by case description of each tumor with regards to molecular subtype, pathology, ploidy level, chromosomal aberrations, chromosomal breakpoints, and gene mutations are given in Supplementary File 2.

### Recurring metachronous urothelial carcinomas show stable genomic alterations

**Figure 2:**
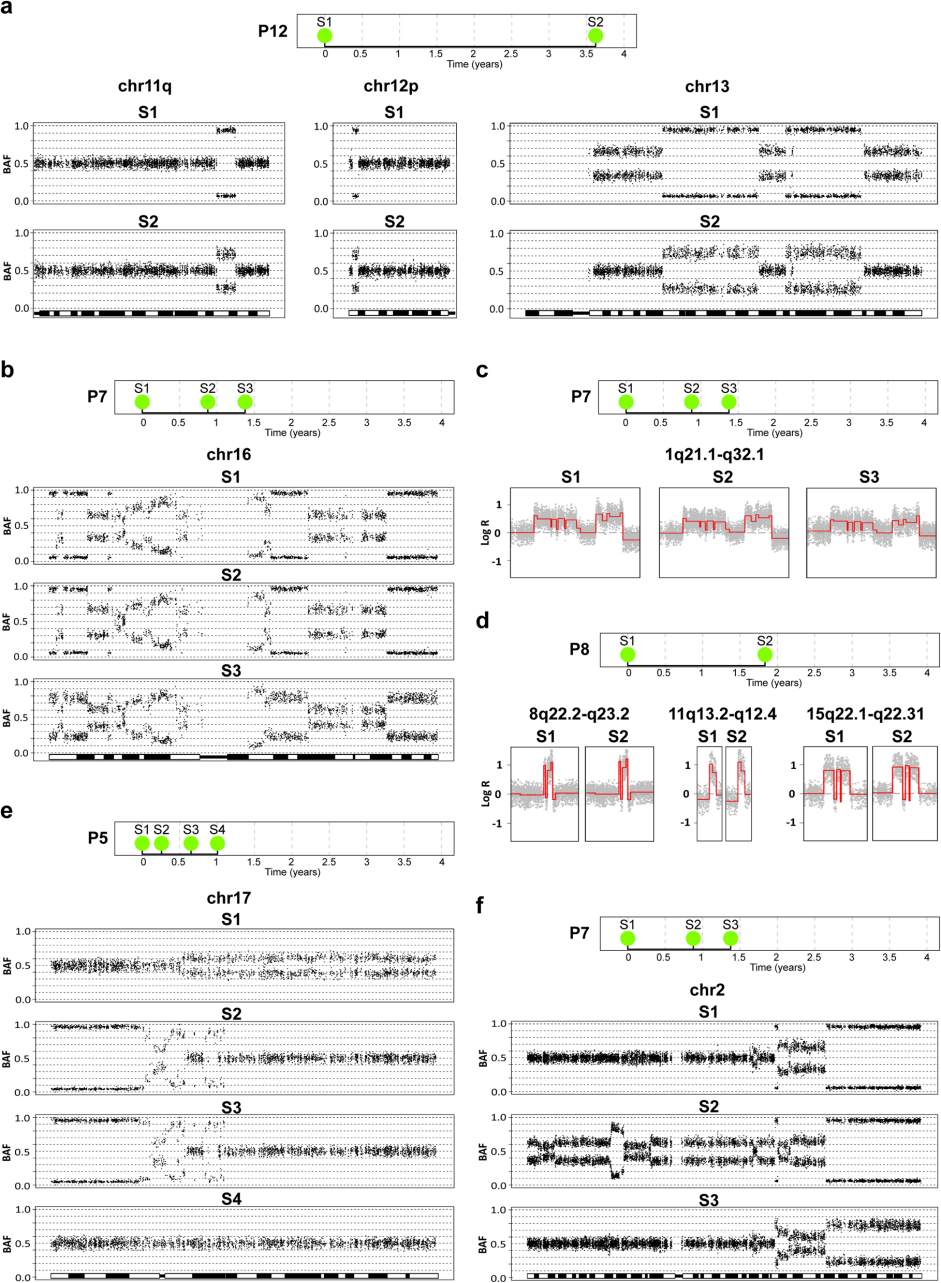
Examples of genomic alterations in metachronous tumors. (**a**) Shared and stable deletions in metachronous tumors. (**b**) Shared and stable complex allelic imbalances in metachronous tumors. (**c**,**d**) Stable complex amplifications as shown by their LRR profiles. (**e**,**f**) Private complex aberrations in metachronous tumors where sudden aberrations may appear in a recurrent tumor and disappear in the following recurrent tumors. All plots except C and D show the B-allele frequencies (BAF). S1, S2, S3, and S4 represent the chronological order of the samples. Red lines, segmented Log R Ratio (LRR). Figure was created in R v3.6 (https://www.r-project.org/) using the packages BAFsegmentation v1.2.0 (https://baseplugins.thep.lu.se/wiki/se.lu.onk.BAFsegmentation) and copynumber v1.28.0 (https://bioconductor.org/).

**Figure 3:**
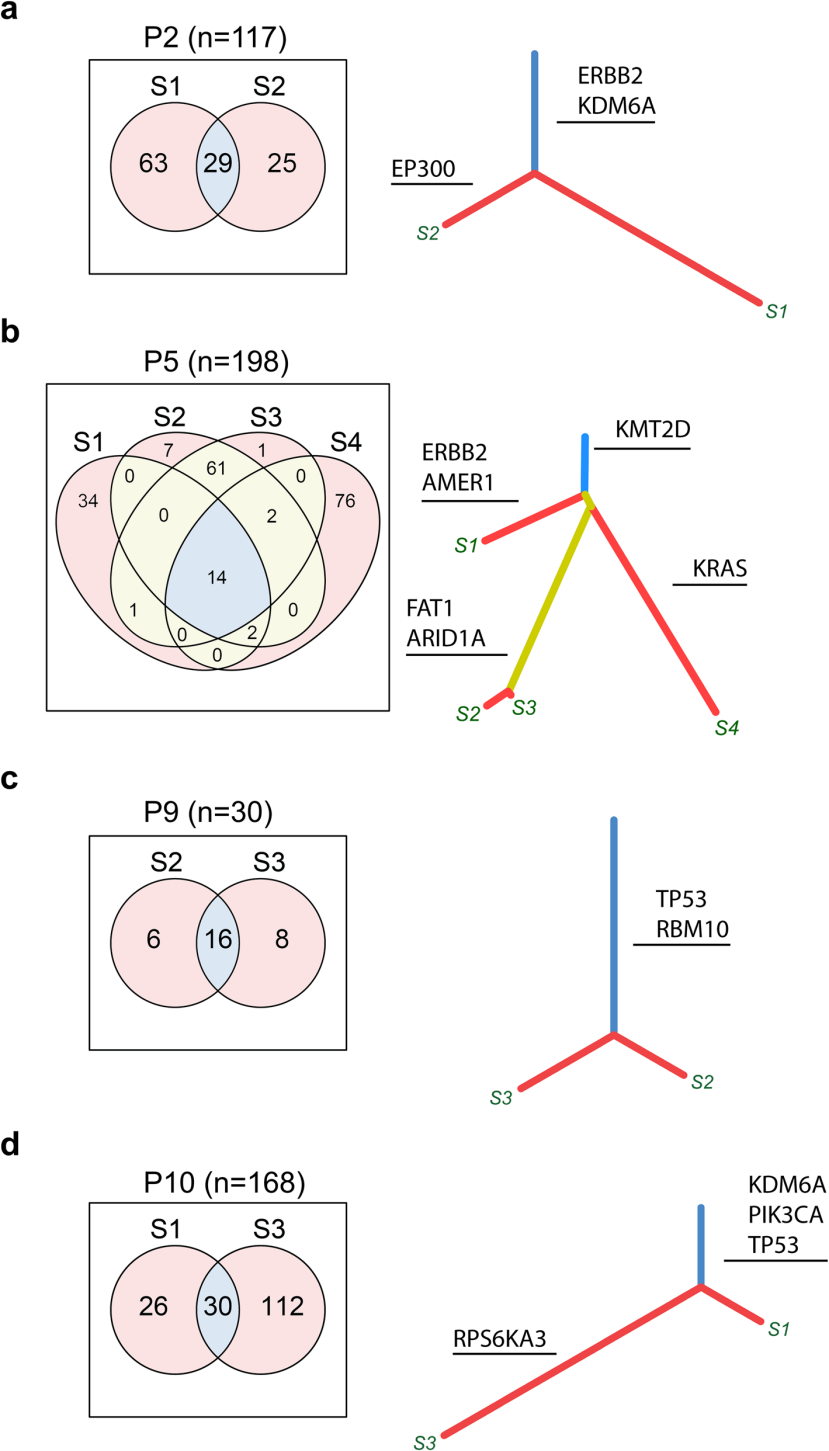
Shared and private mutations in metachronous tumors. The mutations for selected cases (**a**) P2, (**b**) P5, (**c**) P9, and (**d**) P10 are presented in Venn diagrams that show the number of the shared mutations (blue area) and the private mutations in each sample (red area). The yellow area represents mutations shared between subset of tumors from the patient. Phylogenic trees show the mutational data and the lengths represent the number of the mutation in the respective sample. Identified driver genes are indicated in bold. Detailed information on identified gene mutations may be found in Supplementary File 1. Figure was created using R v3.6 (https://www.r-project.org/).

All metachronous tumors from the same patient shared identical breakpoints, ranging from 2 to 105 in numbers. In addition, all metachronous tumors from the same patient shared at least one identical segmental loss as determined by B allele frequency (BAF) values and chromosomal breakpoints. The range of shared losses was 1–10. The single case with only one shared loss had lost the same homolog of chromosome 9, as determined by haplotype in the respective tumors (Supplementary File 2, P4, page 10). Segmental losses also demonstrated long term stability as exemplified by the deletions in 11q, 12p, and 13q sampled as long as 3.5 years apart in patient P12 (Fig. 2a). Also, homozygous deletions were indistinguishable in recurring tumors, as may be seen by the identical deletions in 9p21.3 (*CDKN2A*) in the two tumors from patient P6 collected one year apart (Supplementary File 2, P6, page 13). Highly complex rearrangement involving both gains and losses could show a remarkable stability as seen by the complex pattern of allelic imbalance on chromosome 16 in patient P7 maintained in three recurring tumors (Fig. 2b). Eight of twelve patients with metachronous tumors shared identical gains in all samples. The number of shared gains ranged from 1 to 7 (Supplementary File 2, P1–12). Even in the case of complex amplifications the rearrangements were identical, as exemplified by case P7 in which a complex rearrangement in 1q was present in all three tumors sampled within a two-year period (Fig. 2c). Case P8 harbored three identical genomic amplification in tumors sampled almost two years apart (Fig. 2d). The metachronous tumors from patient P11 collected as much as 2.5 years apart showed similar genomic profiles with gains of 1q and 17q24–25, losses of chromosome 9 and 17p11–13, and a copy neutral loss of heterozygosity at 4p13–16 (Supplementary File 2, P11, page 23). The most frequent clonal losses among the metachronous tumors was deletions on chromosome 9; the loss of whole chromosome 9 in 10 patients, of which two had an additional clonal homozygous deletion at 9p21.3 (*CDKN2A*) and one additional patient with a clonal LOH in 9p21.1–p13.3 (*CDKN2A*). Clonal losses in 8p23.3–p21.3 and 13q12.3–q14.3 (*RB1*) were seen in four patients each. Clonal gains and amplifications were less frequent but gains/amplifications in 1q23.1–q25.1, 3p26.2–p24.3, 8q22.2–q22.3, 17q25.1–q25.3, 20q13.12, were observed in two patients each. The ploidy levels were estimated for all metachronous tumors. In 7 out of 12 patients (P1, P3, P4, P5, P6, P11, and P12) all sampled tumors from the same patient had diploid genomes. In two patients, P7 and P8, all sampled tumors had triploid genomes. The tumors in patients P2, P9, and P10 were polyploid with complex genomes and with no clearly indicated ploidy level in any of the sampled tumors. Examples of the diploid, triploid, and complex polyploid cases are shown in Supplementary Fig. 1 as Log R Ratio (LRR) vs allelic imbalance (AI) plots. All analyzed metachronous tumors from the same patient shared gene mutations, ranging in numbers from 7 to 30 (Supplementary File 1) (Fig. 3). We used the gene mutation data to test for clonality by applying the R-package “Clonality”; all pairwise comparisons showed significant values for clonality (*p* < 0.0001). We then evaluated the shared mutations for potential driver mutations using the principles of Reiter et al.^11^ (Supplementary File 1). In general, metachronous tumors from the same patient showed 1–3 identical driver mutations present in all tumor samples. The most frequently mutated driver genes were *PIK3CA* (4 patients), *FGFR3* (3 patients), and *KDM6A* (3 patients). Taken together, complex genomic alterations, ploidy, as well as driver gene mutations may be stable for several years and appear as identical aberrations in recurring metachronous tumors from the same patient.

### Recurring tumors show non clonal complex changes

In contrast to gross structural alterations stable over time, we also noted examples of complex genomic alterations, not present in all samples from the same patient. In case P5 a complex amplification in 17q was detected in tumors 2 and 3, undetectable in tumors 1 and 4 (Fig. 2e). In case P7, distal chromosome 2 demonstrated complex genomic rearrangements in the first tumor. In the second tumor the p-arm had acquired additional rearrangement, while in the third tumor chromosome 2 was identical to the one observed in the first tumor (Fig. 2f). Chromosome 6 showed two different genomic gains/amplifications in two of the three sampled tumors from case P7 (Supplementary File 2, P7, page 16), and in patient P10 chromosome 3 showed three different patterns of aberrations in the three sampled tumors (Supplementary File 2, P10, page 22). We conclude that complex genomic alterations may be observed in recurrent tumors only to be undetectable in later tumors.

### Individual metachronous tumors from the same patient acquire private changes

We termed breakpoints, genomic rearrangements, and gene mutations private if these were detected in only one of the sampled tumors from a given patient. Tumors from all but two patients (P3 and P8) showed private breakpoints. In total, 22 out of 29 metachronous tumors (76%) demonstrated private chromosomal aberrations. These could be small amplifications or deletions in the mega base pair range, or large and complex events such as depicted in Fig. 2f. The numbers ranged from 1 to 28 for each tumor. In addition, all except one metachronous tumor showed private gene mutations ranging in numbers from 1 to 112 (Fig. 3, Supplementary File 1). Data for private aberrations, chromosomal breaks and for gene mutations data are given for each case in Supplementary File 2. Taken together, even though tumors share many genomic imbalances, chromosomal breaks, and gene mutations, they also acquire private aberrations at all three levels.

### Recurring metachronous tumors show aberrations incompatible with direct relationship

**Figure 4:**
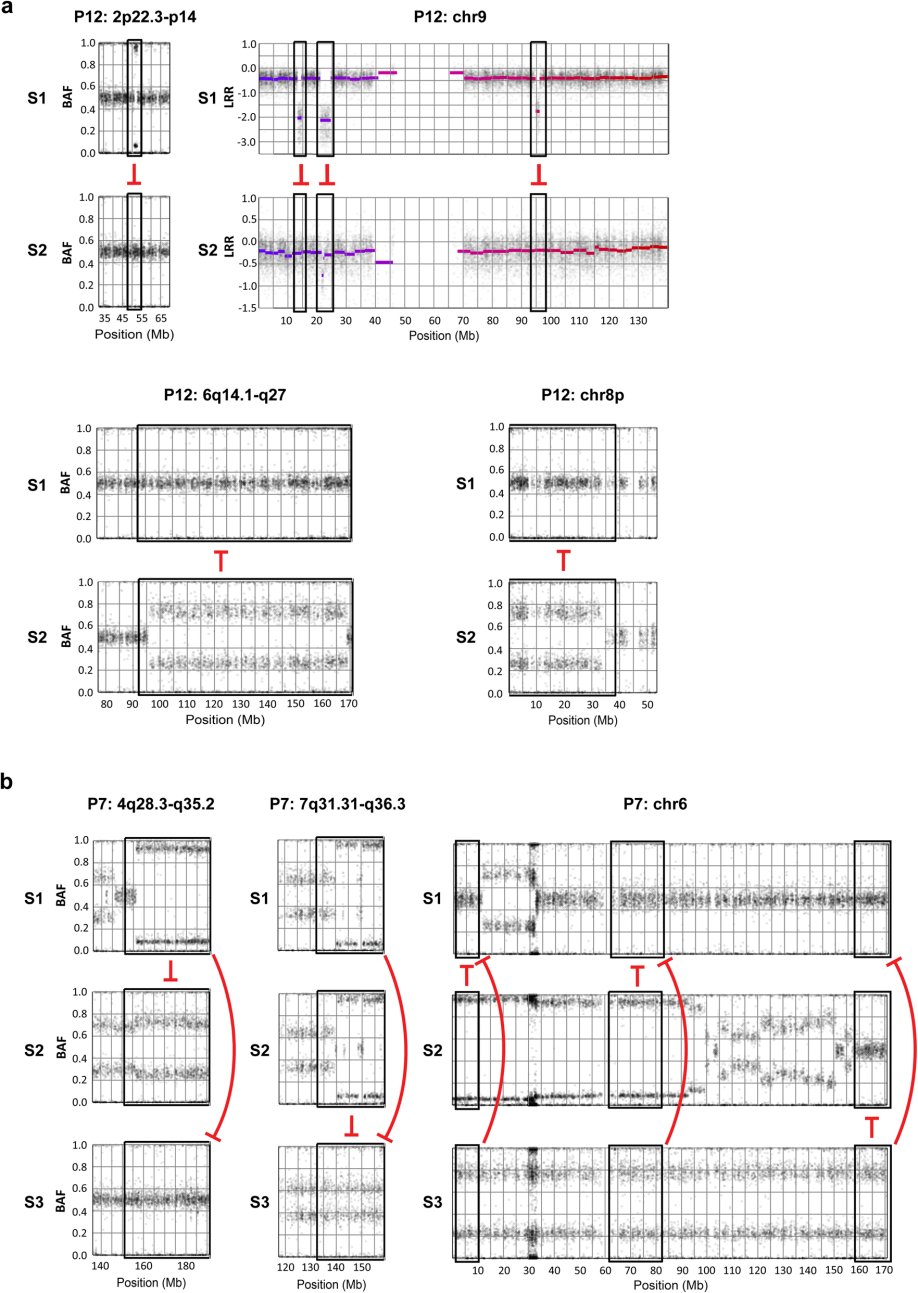
Recurring metachronous tumors show aberrations incompatible with direct relationship. (**a**) A typical example is shown for case P12 in which one distinct AI on chromosome 2p and three on chromosome 9 makes tumor P12_S1 incompatible with being the origin of tumor P12_S2. This incompatibility is reciprocal as the allelic losses on chromosomes 6q and 8p in tumor P12_S2 cannot be regained in tumors P12_S1. (**b**) Tumors P7_S1 shows an LOH in distal 4q (haplotypes A, A,) tumor P7_S2 show a gain of the region (haplotypes A, A, A, B) and tumor P7_S3 has a gain of the same region with the haplotypes A, A, B, B. This makes the first tumor P7_S1 incompatible with the two following tumors. Tumors P7_S1 and P7_S2 shared a loss on arm 7q making them incompatible with P7_S3. Tumors P7_S3, and P7_S2 shows an allelic imbalance in distal 6p as well as in proximal 6q, making it incompatible with tumor P7_S1, and tumor P7_S3 shows an allelic imbalance of distal 6q making it incompatible with tumors P7_S1 and P7_S2. Figure was created in R v3.6 (https://www.r-project.org/) using TAPS v2 package (https://patchwork.r-forge.r-project.org/).

Having established that metachronous tumors displayed both shared stable and private genomic rearrangements we next focused on aberrations defined as *incompatible* with the origin of a second tumor from the previous tumor of the same patient. Archetypal incompatible aberrations are homozygous deletion and loss of heterozygosity, as these changes cannot be reverted in a following tumor. A typical example is shown for case P12 in Fig. 4a in which four distinct homozygous deletions present in S1 was absent in S2 (one on chromosome 2p and three on chromosome 9), unequivocally showing that tumor S1 cannot be the origin of tumor S2. This incompatibility is *reciprocal* as the allelic losses on chromosomes 6q and 8p in tumor S2 cannot be regained in tumor S1, even though the two tumors share many common aberrations. Apart from these changes, tumor S1 showed additional losses at 4p and 11p, not compatible with being the origin of S2, and S2 showed additional losses at 4q, 6q, and 18p not compatible with being the origin of S1 (Supplementary File 2, P12, page 26). In Fig. 4b, a similar analysis for the three metachronous tumors in case P7 is shown. Tumors S1 show a loss of heterozygosity on distal 4q making it incompatible with being the ancestor to the recurring tumors S2 and S3. In addition, tumors S1 and S2 shared a loss on arm 7q making them incompatible with S3. Tumors S2 and S3 show an allelic imbalance in distal 6p as well as in proximal 6q, making them incompatible with tumor S1, and tumor S3 shows an additional allelic imbalance of distal 6q making it incompatible with tumors S1 and S2 (Fig. 4b). Hence, the metachronous tumors in cases P7 and P12 show a number of aberrations that makes them incompatible with being the origin of any of the other tumors from the same patient.

**Figure 5:**
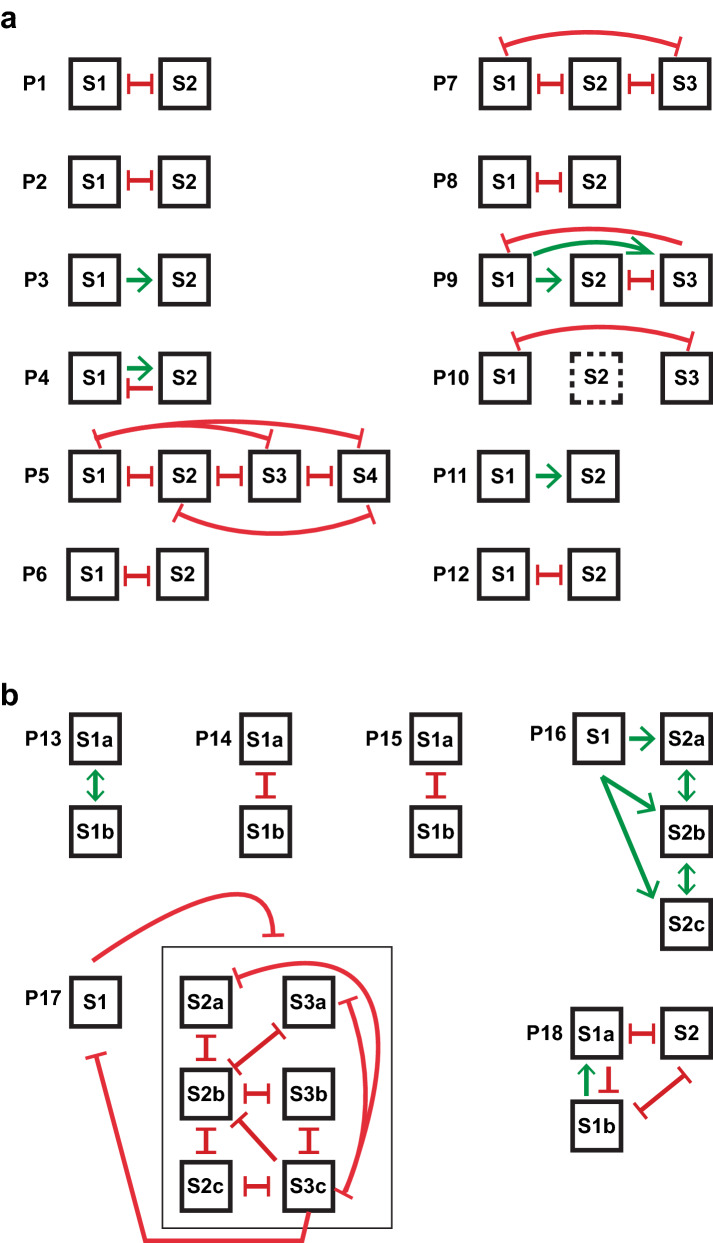
The incompatible genomic relationship between (**a**) metachronous and (**b**) synchronous tumors from the same patient. Truncated red line indicates incompatible tumor relationship. Green arrow indicates a possible direct relationship due to absence of incompatible events. Copy number data was used to determine the incompatibility between samples. Mutation data was used when available. Sample P10_S2 has no clear copy number data or mutation data so no clear conclusion could be drawn for this sample (dotted box). For case P17, green arrows were not shown to reduce the complexity of the figure. Figure was created using Adobe illustrator CS6 v16.0.0 (https://www.adobe.com).

In Fig. 5a we show schematically the incompatible relationships based on genomic aberrations and/or mutation data of tumors from individual patients. Of the 12 patients with metachronous tumors, 7 showed reciprocal incompatibility between all analyzed tumors (patients P1, P2, P5, P6, P7, P8, and P12), tumors from four patients cases showed a pattern compatible with chronological relationship (P3, P4, P9, and P11). For the cases P2 and P10 incompatibility was determined by gene mutation profiles only. Tumors P2_S1 and S2 demonstrated 63 and 25 private mutations, respectively, and shared 29 mutations. Tumors P10_S1 and S3 demonstrated 26 and 112 private mutations, respectively, and shared 30 mutations (Fig. 3). Even though gene mutation data alone cannot formally indicate incompatibility, the large number of private and shared mutations may serve to illustrate a likely genomic evolution and relationship. We conclude that most metachronous tumors, apart from shared genomic alterations indicating a common origin, also display alterations that are incompatible with a successive evolution from a previously occurring tumor; out of 23 possible transitions to a second tumor, 16 (70%) were not compatible with the aberrations present in the preceding tumor.

### Synchronous tumors

We next analyzed genomic data extracted from synchronous tumors to investigate whether they conform to the same evolutionary pattern as the metachronous tumors. Synchronous tumors grow in parallel from the same urothelium and patients thus show multiple tumors. The two synchronous tumors from patient P13, both low grade (G1, G2) Ta tumors of the Lund taxonomy subtype Uro, were both diploid and demonstrated only one chromosomal aberration, a copy neutral loss of 9p (Supplementary File 2, P13, page 28). Patient P16 included one metachronous tumor followed by three synchronous Ta G1/G2 tumors all of molecular subtype Uro. All tumors were diploid and shared a homozygous deletion in 9p21.3 including *CDKN2A*, a gain of 8p, and copy neutral losses of chr9 and 6p25–p21. These two patients restate previous findings that synchronous tumors are not necessarily associated with a high pathological grade and aggressive growth.

Case P14 contained two synchronous GU tumors that shared a gain/amplification of 8q12.1–q24.3 and losses of 5q and 13q12–q21. Apart from this, their genomic profiles differed to a great extent. Tumor S1a contained at least five allelic imbalances at 7p22–p21, 11q23–q23, 15q13–q15, 20q11, and 22q13–q13, respectively, incompatible with S1b. The tumor S1b showed at least five losses incompatible with S1a, at chromosome 4, 8p23–p21, 10q, 18q, and 22q. Apart from this, each tumor showed private and heavily rearrangements on chromosomes 4, 6, 7p, and 8p. The incompatible relationships among tumors from patient P14 are summarized in Fig. 5b. The two synchronous tumors in case P15 shared identical gains/amplifications at 14q11 and 20q13, and both tumors had triploid genomes. However, copy-neutral loss of heterozygosity on 11q in S1a and on 2p21–p25.3 in P15_S1b make these two tumors incompatible with each other (Fig. 5b).

In case P17, one metachronous tumor was followed by three synchronous tumors after six months and three additional synchronous tumors six months later. These tumors were all diploid and shared a copy neutral LOH of chromosome 9 and a small deletion on 2q37. The tumors also shared 23 gene mutations, including driver mutations in *CDKN2A* and *PIK3CA.* All six synchronous tumors had obtained a further mutation in *DLL4* as well as an amplification at 11q22–q22, located in a highly rearranged chromosome 11 (Supplementary File 2, P17, page 34). Tumors S2a, 2b, and 2c differed by gaining one additional private gene mutation each. Tumor S3a gained three private mutations, whereas S3b and 3c did not acquire any further mutations. All six synchronous tumors differed from the preceding single tumor by showing identical losses of 5q11–q14, 7p22–p12, 8p, and 10q as well as gains of chromosome 19 (Supplementary File 2, P17, page 35). In spite of the great similarity at the gene mutation and genomic levels, the seven tumors showed an intricate net of incompatible aberrations. The first metachronous tumor S1 showed a small region of allelic imbalance in 11q14, incompatible with all the subsequent synchronous tumors. Chromosome 11 also harbored a region with allelic loss in proximal 11p in all cases except in S2a and S3c. Tumor S3c contained allelic losses at 13q14.11–q34, 17p11.2–p13.3, and at 20p12.1 incompatible with all other tumors (Supplementary File 2, P17, page 35). The incompatible relationships among these seven tumors are summarized in Fig. 5b.

Case P18 presented with two synchronous Uro tumors followed by a single recurrent Uro tumor one and a half year later. The tumors were clonally related as all three shared four identical genomic aberration; gain of 3p22–26, and losses at 4q31–q35, 6q14–q22, and 13q12–14. One of the synchronous tumors, S1a, contained three losses at 3p14, 4q22 and 7q11, incompatible with the synchronous tumor S1b and the recurring single tumor S2. Furthermore, tumor S1b harbored 8 gene mutations not detected in S2. No mutation data was available for S1a. The incompatible relationships for P18 are summarized in Fig. 5b. Taken together, even if synchronous tumors appear in parallel, in the same diseased urothelium, and do share genomic alterations, they may show additional incompatible genomic or gene mutation events that makes it impossible to have evolved from each other directly.

### Molecular subtype shows remarkable stability in recurring tumors

All tumors were classified molecularly according to the Lund classifications system using gene expression analysis (Fig. 1). The majority (38 out of 45 tumors) were of the Urothelial-like (Uro) subtype, five were classified as Genomically Unstable (GU), and two as Basal/Squamous-like (Ba/Sq). No shifts in molecular subtype occurred among the metachronous tumors from the same patient. Even in patient P5 from which four different recurrences were sampled, and in which all tumors showed reciprocal incompatible genomic events, all tumors were of the same molecular subtype (Figs. 1 and 5, and Supplementary File 2, P5, page 11). All synchronous tumors from the same patient were of the same molecular subtype. In four patients all tumors were Uro, and in one patient all were GU. In case P17, from which six tumors were classified, all were of the Uro subtype (Fig. 1), even if the majority showed reciprocal incompatible genomic events (Fig. 5 and Supplementary File 2, P17, page 35). In conclusion, a given urothelium seems to initiate recurring tumors with the same molecular subtype without being affected by the observed genomic instability and variation.

## Discussion

Patients with non-muscle invasive urothelial carcinoma are characterized by having numerous recurrences after transurethral resection (TUR). Patients may also show multiple tumors, sometimes far apart within the bladder wall. To gain insight into the origins and dynamics of urothelial carcinoma several studies have analyzed the adjoining tumor free urothelium for genomic and gene alterations. The most detailed genetic studies of the premalignant urothelium in patients with UC has been performed by the groups of Dyrskjøt^9,10^ and Czerniak^11,12^, showing that the urothelium in patients with urothelial carcinomas are replete with changes also present in the overt tumors. We instead analyzed several meta- and synchronous tumors from the same patient, as each overt tumor may be considered a clonal expansion of the underlying urothelium. This approach allows for an analysis of the evolutionary relationship between tumors recurring in the same genetic background. The first conclusion made was that all tumors from a given patient displayed clonal features. This was shown by the sharing of sometimes complex genomic rearrangements, gene mutations, as well as chromosomal breakpoint patterns in temporally distinct tumors. In some cases, highly complex changes were stable in every detail and appeared in recurring tumors during several years. Furthermore, recurring tumors shared many identical gene mutations, including mutations in driver genes such as *FGFR3*, *PIK3CA*, and *KDM6A.* The observed genomic stability also included the ploidy level as no ploidy changes were observed in tumors from individual patients. Importantly, this does not only demonstrate clonal relationships but also show that specific chromosome alterations and gene mutations must have been present in the urothelium in periods when patients were considered tumor free. Hence, the premalignant urothelium in these patients must harbor stable complex genomic rearrangements as well as mutations in possible driver genes.

In some patients, very complex genomic aberrations were observed in recurring tumors that was not detected in the previous tumor. These gross structural changes could be observed as identically rearranged chromosomes in a later recurrence, only to be absent in a subsequent recurrence. Within the window of our analyses, these changes occurred with no signs of a stepwise increase or decrease in complexity. Hence, when local genomic alterations have occurred, they may be identical in a recurring tumor, only to be undetectable in a later recurrence. We used the term “private changes” for aberrations only present in one of the sampled tumors. The sheer numbers of observed private aberration in individual tumors clearly demonstrate an ongoing divergence before overt tumor formation. This divergence will produce an increase in genomic complexity that may lead to the appearance of what we termed *incompatible* events, essentially non-reversible unique chromosomal changes, e.g., homozygous losses or loss of heterozygosity, incompatible with one tumor being the origin of another. By systematically identifying incompatible events we were able to show that these events were not only common, but also that most tumors sampled from the same patient showed *reciprocal incompatibility*, and thus none of the tumors could be the origin of the others. This held true not only for the metachronous tumors but also for synchronous tumors i.e., multiple tumors occurring in parallel in the same urothelium. Hence, even synchronous tumors do not originate from each other. Furthermore, as the observed reciprocal incompatible changes exclude a direct relationship between recurring overt tumors, it also excludes seeding and incomplete resection as a mechanism for tumor spread and re-initiation. Thus, our findings argue against a parent–child relationship among meta- and synchronous UC tumors and instead suggest a sibling relationship; they share both common genetic features and demonstrate unique non-reversible genetic features. This model still holds for the relationship among recurring tumors that do not show incompatible aberrations, however, in these situations seeding and other mechanisms cannot be excluded.

**Figure 6:**
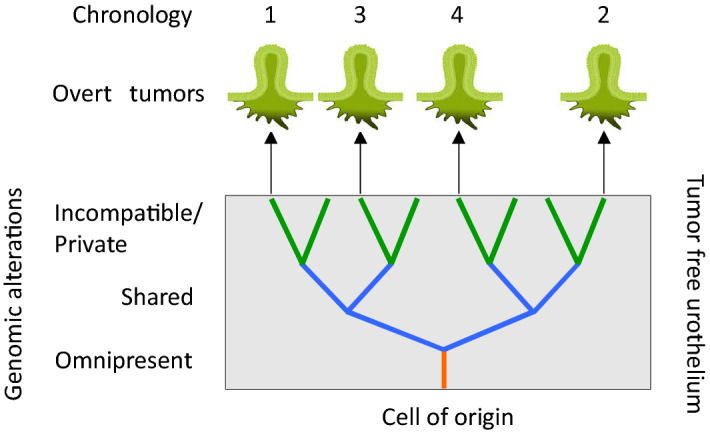
Schematic presentation of the findings. A diseased and hard wired urothelium destined to produce tumors of a given molecular subtype (green). The branching tree indicates an ongoing genomic evolution and spread of the original clone in the tumor free urothelium. Four metachronous tumors originate from different branches of the three. The tumors display omnipresent aberrations (orange) i.e., genomic aberrations present in all metachronous tumors from the same patient; shared aberrations (blue), present in some but not all metachronous tumors; private/incompatible aberrations (green), present in single metachronous tumors, i.e. aberrations that prohibit the parenthood to another metachronous tumor lacking a specific aberration. Chronology, the order at which the overt tumors appeared. The schematic image applies equally well to synchronous tumors by excluding the chronology. Scheme was created using BioRender (https://biorender.com/) and Microsoft PowerPoint 2019 (https://www.microsoft.com).

One approach to understand the presence of stable complex genomic rearrangements and driver gene mutations in the urothelium without apparent tumor growth is to compare the genomically unstable urothelium with a decaying complex genetic system. Genetic systems are known to be robust and to show several alternative routes for information transfer^13^, such as functional redundancy i.e., genes with overlapping functions resulting in similar effects that ensures robustness against loss of function mutations. Hence, stochastic interruptions of elements and links may be tolerated with a maintained systemic function, as has been shown to be the case for complex network systems^14^. However, the decaying process will eventually push the system towards a state of criticality in which previously trivial changes in this new context may drive the system over a tipping point and induce local transformation. Consequently, the inducing factors may have no impact in the original system but have a strong impact in a context of a decaying system. This could explain why driver mutations may be present in the evolving urothelium without the growth of overt tumors, and why, if and when a patient will have recurrence after tumor resection is hard to predict; in the end this depends on chance events. It also suggests that driver mutations, at least in the case of urothelial carcinoma, may execute their main functions during the premalignant stage. In this respect urothelial carcinoma behaves like a two-phase disease with periods of a genomically diverging but tumor free urothelium^3^, interrupted by bursts of active tumor growth^15^, as mushrooms from an underlying mycelium (Fig. 6). This may also explain the noted weak association between chronological appearance of tumors and their genomic complexity^16^; recurring tumors may be initiated from different branches of the genomic evolution tree operating in the urothelium (Fig. 6). Still, the size and the genetic heterogeneity of the field will most likely affect the likelihood for recurrences as genetic heterogeneity has been found to be predictive of premalignant lesions in Barrett’s esophagus^17^ and of breast cancer progression^18^.

Even though the genomes of the precancerous urothelial cells in patients with recurring tumors seems to be highly dynamic, the overt tumors from the same patient invariably were of the same molecular subtype. In a recent investigation we analyzed tumors from a large number of patients with a considerable number of recurrences both by gene expression and by IHC and concluded that patients had a strong tendency to recur with tumors of the same molecular subtype^19^. No comprehensive genomic analyses were performed in this series, however, even when progressing to muscle invasive growth the original molecular subtype was maintained in several cases. This suggests that the urothelium in patients with urothelial carcinomas is “pre-wired” to repeatedly produce a specific molecular subtype and that, in the perspective of the present findings, this process is resilient to the observed genomic instability/variability. This is reminiscent of the maintenance of functional driver-gene profiles on a background of mutations in less essential genes that may differ between primary and metastatic tumors^11^ and relates to the finding that gene mutations are enriched in “non-essential” i.e., non-transcribed, regions of the genome^20^. Translated into the present findings, many chromosomal aberrations would appear in “non-essential” regions of the genome. Seen from this perspective, even if useful for establishing clonal relationships, most of the genomic alterations creating genetic heterogeneity described in the present investigation may be irrelevant for the actual nature of the tumors. To summarize, our data suggest a model for the origin of recurring tumors that emphasize a genomically destabilized but tumor free urothelium that may be subjected to extensive genomic evolution without the appearance of overt tumors. Our data show that most recurring tumors do not originate from a previous overt tumor but rather from a shared field of diseased but tumor free urothelium. This shift the focus on why and when a patient recurs with a new tumor from the nature of the previous tumor to nature of the field of diseased cells.

## Materials and methods

### Patient material

The exophytic parts of urothelial tumors were collected by cold-cup biopsies from patients undergoing transurethral resection at the Lund University Hospital, Sweden, between 2001 and 2007. In total, 49 tumors from 18 patients and 18 matched normal blood samples were collected. The study was approved by the Lund University ethics committee (LU 51-01) and informed consent was obtained from all patients. The methods employed were according to the guidelines and regulations afforded by the Lund University ethics committee. Detailed sample characteristics are presented in Supplementary Table 1. Tumors were labeled as *synchronous* if they were resected at the same timepoint from patients with multiple tumors, otherwise as *metachronous*.

### DNA and RNA isolation

Genomic DNA was isolated from frozen tumor tissue using the DNeasy Blood and Tissue Kit (Qiagen, Valencia, CA), including the optional RNase H treatment, and verified for high quality using agarose gel electrophoresis. DNA from matched blood samples were isolated using the Gentra Puregene Blood Kit (Qiagen, Valencia, CA).

### Copy number analyses

Genomic DNA from tumor and matched normal blood samples were hybridized on HumanCNV370 Genotyping BeadChips (Illumina Inc., San Diego, CA) at the SCIBLU Genomics Centre at Lund University, Sweden according to the manufacturer’s specifications. Raw data signals were extracted and normalized from image files containing florescent signals using the GenomeStudio 2.0 software (Illumina Inc.). To correct for asymmetry in copy number (CN) and B-allele frequency (BAF) due to dye-bias, data was exported from GenomeStudio 2.0 and normalized using transformed quantile normalization, tQN^21^. Normalized CN data was then segmented and visualized using the R packages: BAFsegmentation^22^, TAPS^23^, copynumber^24^, and ASCAT (v2.5)^25^. All the tools were run using the default settings except for BAF segmentation where the size limit for segments was set to at least 10 SNPs. The Log R Ratio (LRR) baseline in all samples was reviewed using CopyNumber450kCancer R package^26^. Manual inspection of all segmented LRR and mirrored BAF (mBAF) profiles were performed. Purity, ploidy, CN events, and sub-clonality were examined as described in Lindgren et al.^27^ and compared between the samples from the same patient when possible. To evaluate and compare the chromosomal events, a detailed haplotype and breakpoint analyses were performed. The breakpoints were defined based on mBAF segments. Breakpoints were compared between samples in each patient. In this comparison, the breakpoints in different samples were labeled as identical if they were located within a window of 100Kbp (~ 3 to 4 probes). If two samples shared the same breakpoints for an event, the haplotype in the two samples were then compared to check if the events were located on the same parental chromosome. In the Illumina assay, SNPs are arbitrarily called A or B. Using the BAF for the SNPs in the CNA region, the haplotype can be extracted. Haplotype is defined here as a consecutive series of A or B showing the same segmented BAF and LRR value. This was only performed on loci that were heterozygous in the matched normal control. Defining the haplotype for the CNAs gives an additional opportunity to investigate clonal relationships between tumors. For example, if two tumors from the same patients where chromosome 9 was lost in both tumors, identical haplotypes means loss of the same chromosome homolog and implies clonal relationship. CTLPScanner^28^ web service was used for chromothripsis-like pattern detection when needed. Sex chromosomes were excluded from all analyses. Segmented CN data is available in Supplementary File 3.

### Mutation analysis

For 27 tumor samples and matched normal blood samples from 10 patients, a custom target captured panel (Agilent SureSelect) was used to sequence 1697 cancer genes^29^. The sequencing was performed according to the manufacturer’s specifications (BGI Technologies). Variant calling was performed by MuTect v1.1.4^30^ and VarScan v2.3.5^31^. MuTect tool was used with default settings. The parameters for VarScan were as following: Tumor frequency ≥ 10%, Tumor reads ≥ 4, Normal frequency < 3%. Only the somatic variants called by the two methods were used in the study. Oncotator^32^ was used to annotate the identified variants. The sequenced genes and the variants in each patient are listed in Supplementary File 1. Gene mutation based Clonality analysis were performed using the R package Clonality^33^. Gene mutation frequency data was obtained from the TCGA bladder cancer data^34^ (n = 411 samples). We used 10,000 permutations to estimate *p* values. Clonality results are listed in Supplementary File 1.

### Identification of putative drive genes

Mutation effect analysis was performed using Likely Functional Driver (LiFD) tool^11^, https://www.github.com/johannesreiter/lifd) using default settings, the results of this analysis is available in Supplementary File 1. LiFD runs in two phases, in the first phase it uses 4 databases (OncoKB, CGI, known cancer hotspots, and COSMIC) and in the second phase it uses 5 prediction tools (CHASMplus, FATHMM, CanDrA, CGI, and VEP). A mutation is labeled as functional if it is present in any of the four databases or if > 50% of the tools predicted it to be a functional mutation.

### Gene expression data

Gene expression data for 47 tumor samples from 18 patients were generated using Illumina HumanHT-12 V3.0/V4.0 Expression BeadChip in the SCIBLU facility at Lund University (https://www.lth.se/sciblu) according to the manufacturer’s specifications. Gene expression data with sample annotations is available at the Gene Expression Omnibus under the accession number (GSE146870). Tumors were classified according the Lund classification system into Urothelial-like (Uro), Genomically Unstable (GU), Basal/Squamous-like (Ba/Sq), Mesenchymal-like (Mes-like), and small cell/neuroendocrine like (Sc/Ne-like) using gene expression data^35^. Final classification of the tumors from patient 9 (P9) was performed by immunohistochemistry^36^.

## Data and code availability

The gene expression data is available publicly on (GSE146870). The mutation data, segmented copy number data and the generated data are available as supplementary materials in this paper. The availability of the raw genotyping arrays data and raw sequencing data is restricted due to the General Data Protection Regulation (GDPR).

## Supplementary information

Supplementary Information 1.

Supplementary Information 2.

Supplementary Information 3.

Supplementary Information 4.
