# Supplementary material for: Recurring urothelial carcinomas show genomic rearrangements incompatible with a direct relationship
Source: Sci Rep. 2020 Nov 11;10:19539. doi: 10.1038/s41598-020-75854-4 (PMC7658206; doi:10.1038/s41598-020-75854-4)
Supplement: Supplementary file 4 — Supplementary Information 4. [file 41598_2020_75854_MOESM4_ESM.pdf]

Recurring urothelial carcinomas show genomic rearrangements incompatible with a direct relationship

Nour-Al-Dain Marzouka, David Lindgren, Pontus Eriksson, Gottfrid Sjödahl, Carina Bernardo, Fredrik Liedberg, Håkan Axelson, Mattias Höglund

Supplementary Information:

- Supplementary Figure 1
- Supplementary File 2

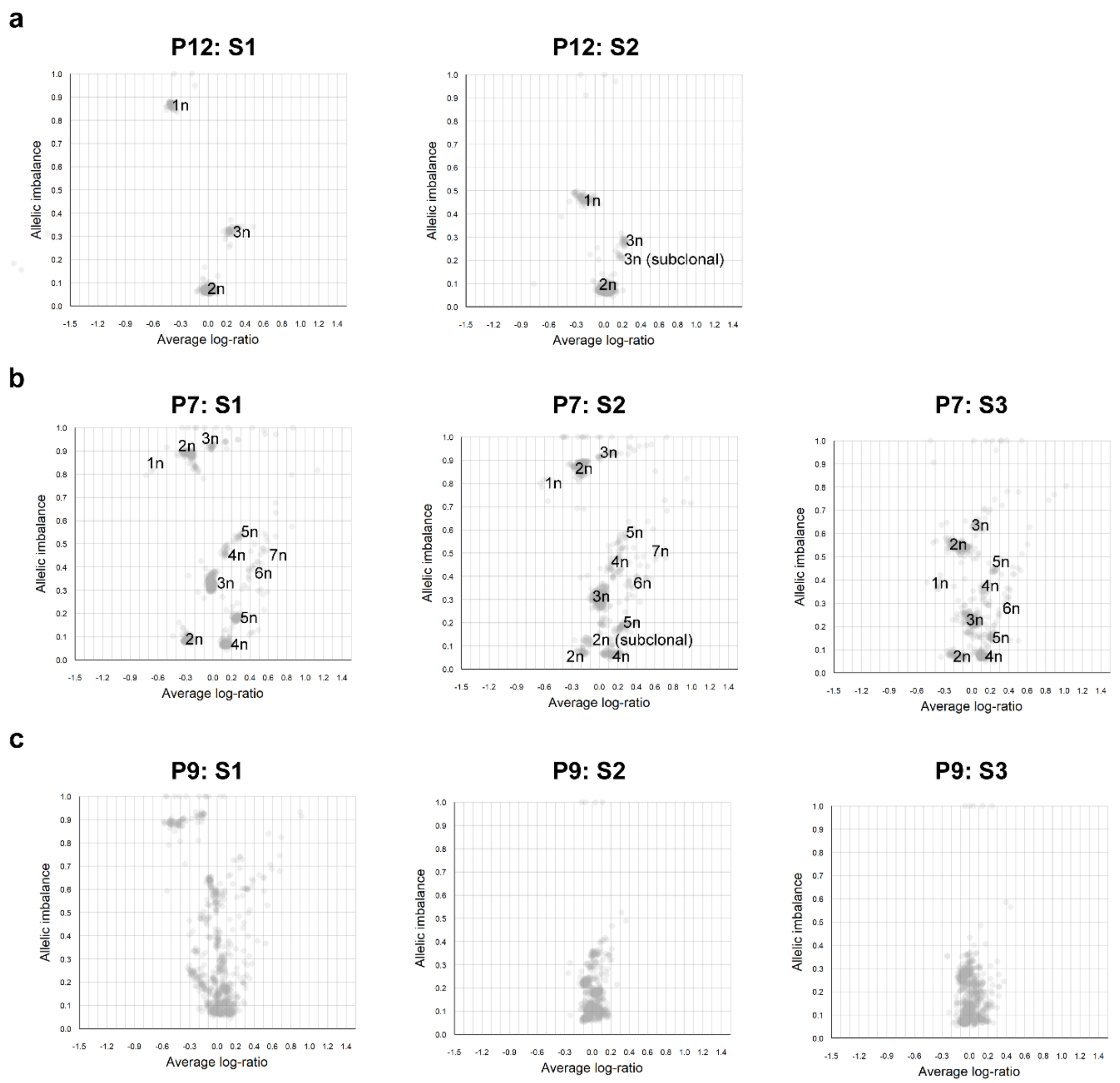

Supplementary Figure 1. Ploidy levels is stable in metachronous tumors. Allelic imbalances (AI) versus Log R Ratio (LRR) plots show the stable ploidy level in tumors from the same patient. **A)** Profile for an example case with tumors have diploid genome. **B)** Profile for an example case with tumors have triploid genome. **C)** Profile for an example case with tumors have complex polyploid genome. Figure was created in R v3.6 (https://www.r-project.org/) using TAPS v2 package (http://patchwork.r-forge.r-project.org/).

Supplementary File 2

P1

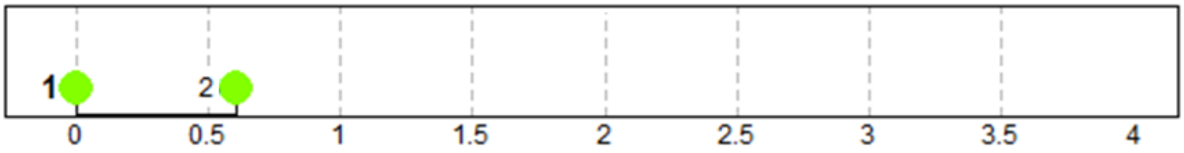

Tumor characteristics:

Sample	Pathology	Molecular subtype	Purity	Ploidy
**P1_S1**	TaG3	Uro[1]	91%	Diploid
**P1_S2**	T1G2	Uro[1]	91%	Diploid

1) Uro: Urothelial-like

Mutation data:

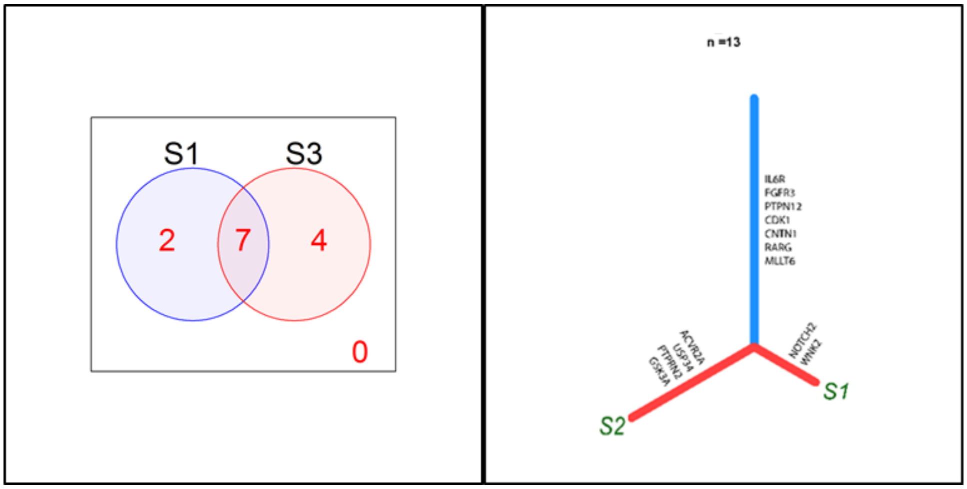

Breakpoints (window size 100K):

	P1_S1	P1_S2
**P1_S1**	**36**	17
**P1_S2**	17	**62**

Shared Imbalances:

Gains: NA

Amplifications: NA

Losses: 6q15, -8p, -chr9, -11p, 12p13.31-p11.1, 12q24.33, 14q22.1-q31.1

Homozygous deletions (HD): NA

Copy-neutral LOH: NA

Private alterations:

Compatible: NA

Incompatible events:

P1_S1: Losses: 17q25.3, 20p13

HD: 8p21.2

P1_S2: Losses: 4q28.3, 20p13

Sub clonal events: NA

Selected incompatible alterations:

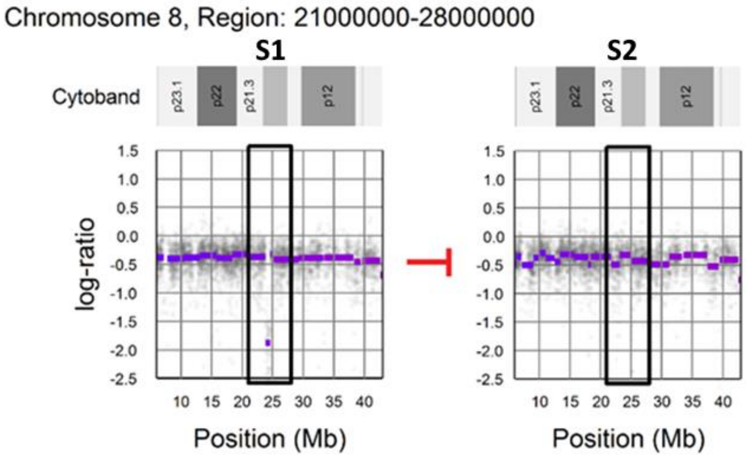

P2

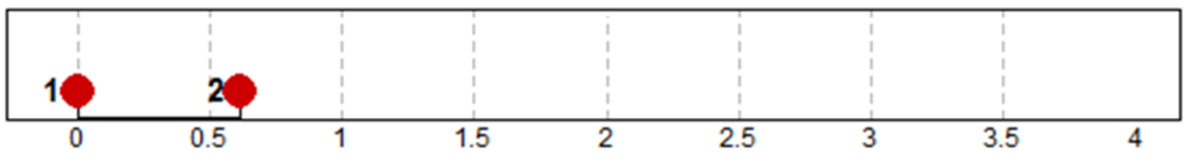

Tumor characteristics:

Sample	Pathology	Molecular subtype	Purity	Ploidy
**P2_S1**	T2G3	Ba/Sq[1]	Very low[2]	Polyploid[3]
**P2_S2**	T2G3	Ba/Sq[1]	Very low[2]	Polyploid[3]

1) Ba/Sq: Basal/Squamous like. 2) Very hard to estimate.

3) Polyploidy but n is undetermined due to sample complexity.

Mutation data:

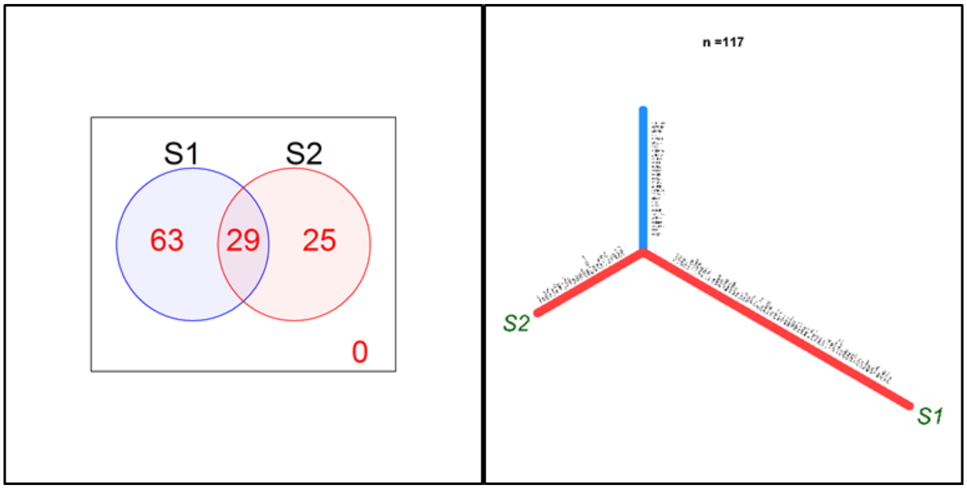

Breakpoints (window size 100K):

	P2_S1	P2_S2
**P2_S1**	**331**	54
**P2_S2**	53	**228**

Shared Imbalances: Not all events can be listed due to the sample complexity and low purity.

Gains: Not listed

Amplifications: 1q23.1-q25.1, 2p16.1-p14, 3p26.2-p24.3, 11p13-p12, 12q14.2-q21.1

Losses: Not listed

Homozygous deletions (HD): Not listed

Copy-neutral LOH: Not listed

Private alterations: Not all events can be listed due to the sample complexity.

Compatible: Not listed

Incompatible events:

P2_S1: No clear incompatible events

P2_S2: No clear incompatible events

Sub clonal events: Not listed

Selected examples of A) shared and B) altered imbalances:

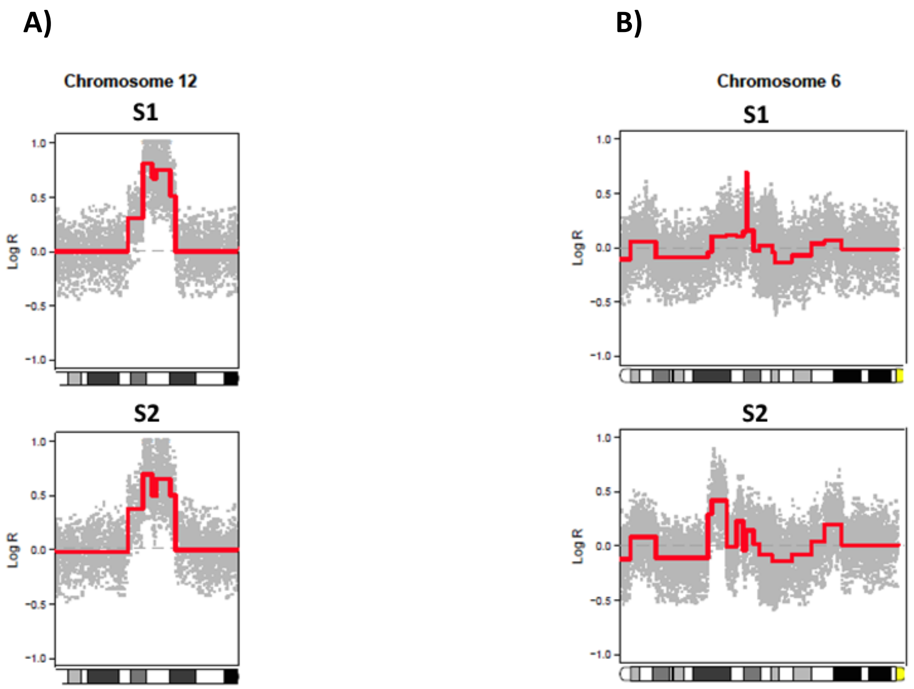

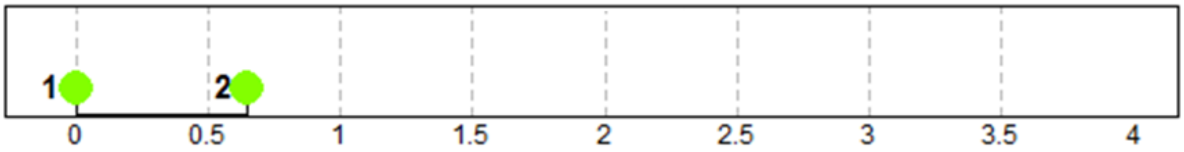

Tumor characteristics:

Sample	Pathology	Molecular subtype	Purity	Ploidy
**P3_S1**	T1G2	Uro	91%	Diploid
**P3_S2**	T1G3	Uro	96%	Diploid

Mutation data: NA

Breakpoints (window size 100K):

	P3_S1	P3_S2
**P3_S1**	**51**	38
**P3_S2**	38	**60**

Shared Imbalances:

Gains: 8p11.21-q24.3,

Amplifications: 8p11.23-p11.21,

Losses: 2q37.1, 8p23.3-p11.23, chr9, 10p15.3, 10p15.1, 10p14-p11.21, 10q21.1, 17p11.1, 17q21.32, 17p13.3-p11.1

Homozygous deletions (HD): NA

Copy-neutral LOH: NA

Chromothripsis: chr21

Private alterations:

Compatible: NA

Incompatible events: NA

Sub clonal events:

P3_S1: Gains: 4q13.1

P3_S2: Gains: 5p14.3

Losses: 3q13.2-3q27.2

Selected shared complex alterations:

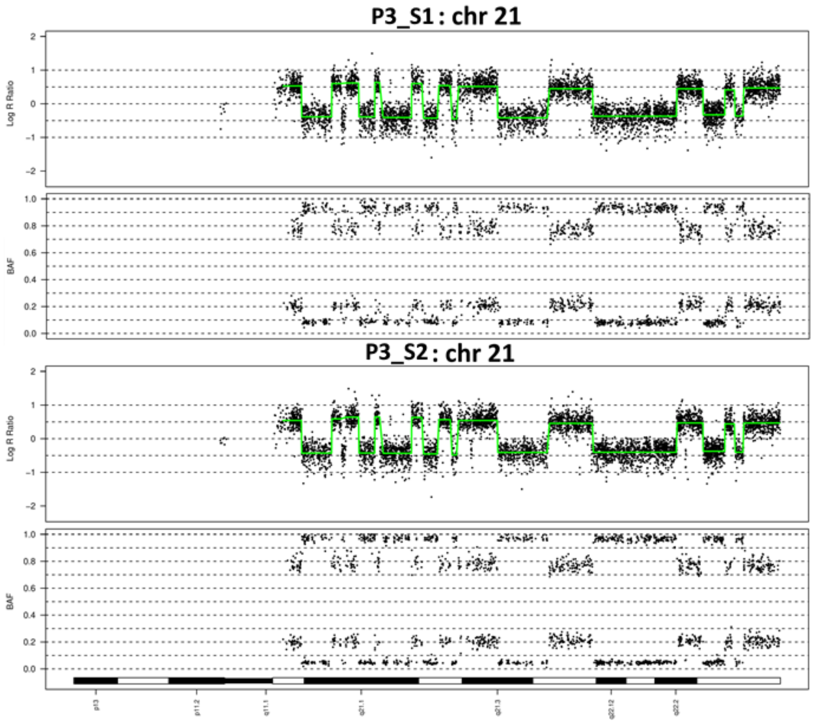

P4

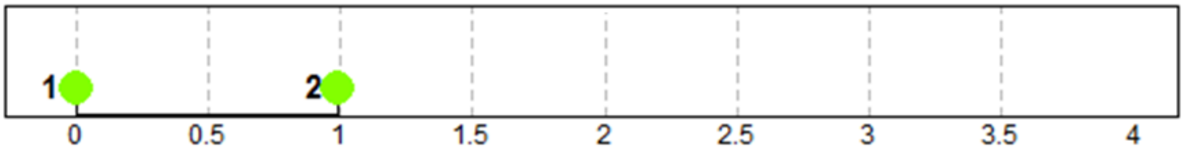

Tumor characteristics:

Sample	Pathology	Molecular subtype	Purity	Ploidy
**P4_S1**	TaG2	Uro	75%	Diploid
**P4_S2**	TaG1	Uro	93%	Diploid

Mutation data:

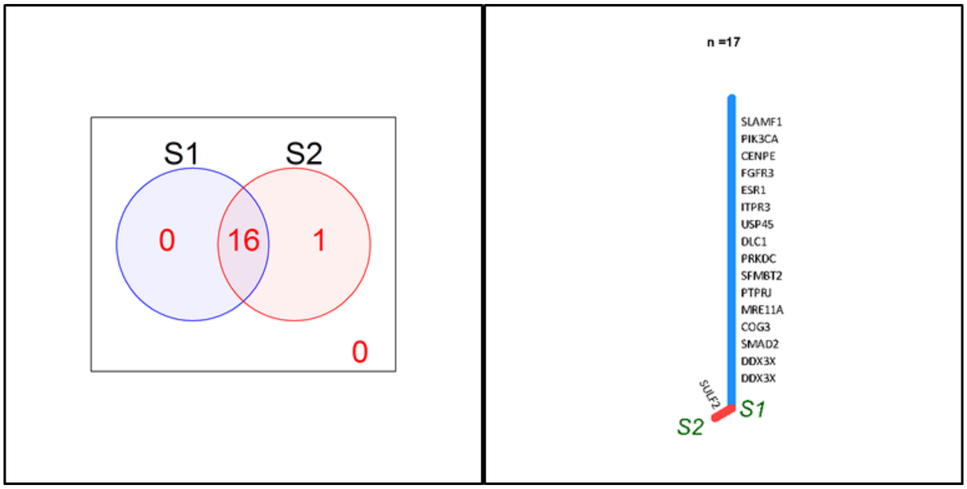

Breakpoints (window size 100K):

	P4_S1	P4_S2
**P4_S1**	**20**	2
**P4_S2**	2	**19**

Shared Imbalances:

Gains: NA

Amplifications: NA

Losses: -chr9

Homozygous deletions (HD): NA

Copy-neutral LOH: NA

Private alterations:

Compatible: NA

Incompatible events:

P4_S1: NA

P4_S2: Losses: 3p24.2-p24.1, 3p21.31-p14.3

HD: 9p21.3

Sub clonal events:

P4_S1: Gains: 1q21.2-1q44

P4_S2: Gains: 1q

Shared deletion of chromosome 9:

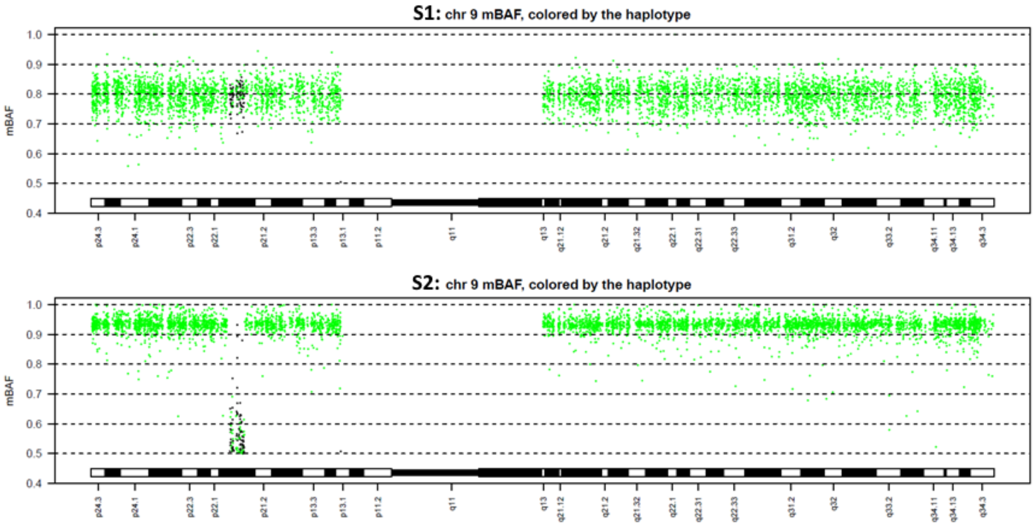

Deletion of one copy of chromosome 9 in both samples. SNPs are colored in green when the allelic imbalance in both samples occurred on the same haplotype. The region with black dots represents a homozygous deletion in P4_S2 but not in P4_S1.

P5

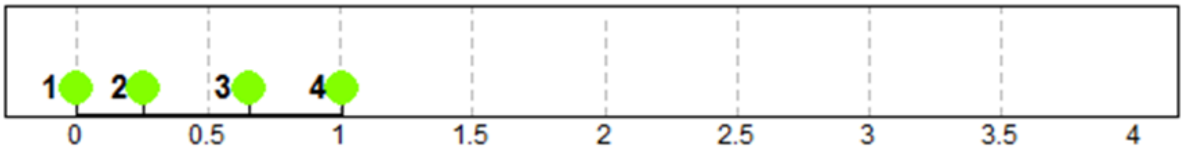

Tumor characteristics:

Sample	Pathology	Molecular subtype	Purity	Ploidy
**P5_S1**	TaG2	Uro	39%	Diploid
**P5_S2**	T1G2	Uro	94%	Diploid
**P5_S3**	T1G3	Uro	91%	Diploid
**P5_S4**	T1G2	Uro	80%	Diploid

Mutation data:

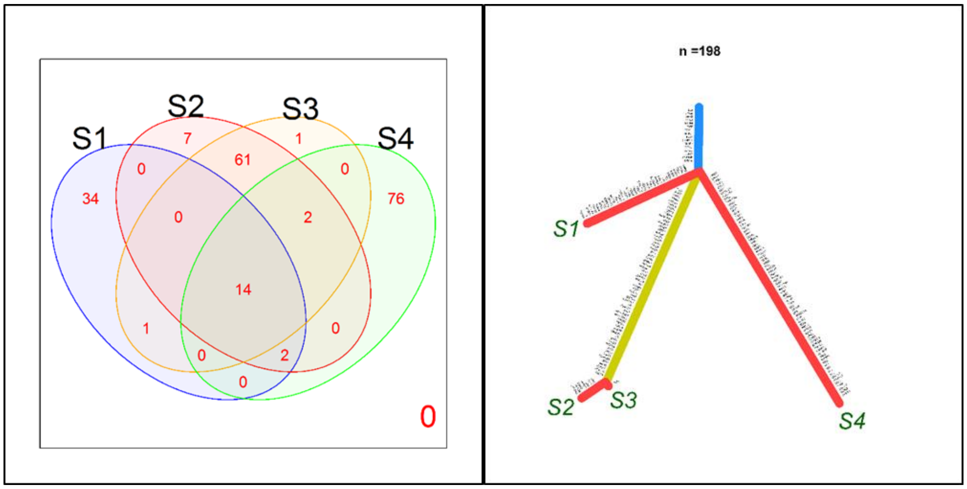

Breakpoints (window size 100K):

	**P5_S1**	**P5_S2**	**P5_S3**	**P5_S4**
**P5_S1**	**41**	9	7	5
**P5_S2**	9	**88**	26	5
**P5_S3**	7	26	**50**	9
**P5_S4**	5	5	9	**40**

Shared Imbalances (The event is present in all samples unless indicated between brackets)**:**

Gains: 8q

Amplifications: NA

Losses: 8p, 8q24.23, 9p23-p22.2

Homozygous deletions (HD): NA

Copy-neutral LOH: NA

Complex event: 17 (in S2 & S3)

Private alterations:

Compatible:

P5_S1, P5_S2, and P5_S4: NA

P5_S3: Gains: 2q33.1, 3p26.3-3p21.1, 15q26.1

Incompatible events (The sample is incompatible with the tumors between brackets**):**

P5_S1: CN LOH: 1p36.12-p34.2 (S2, S3), 4q34.3-q35.2(S2, S3, S4), 17q (S2, S3, S4)

P5_S2: Losses: 2q36.3-q37.3 (S1, S4),
CN LOH: 1p36.33-1p36.12 (S1, S4), 17p (S1 S4, but shared with S3)

P5_S3: Losses: 2q33.1-q37.3 (S1, S2, S4), 18q12.2-q23 (S1, S2, S4)
CN LOH: 1p36.33-p36.12 (S1, S4), 17p (S1, S4, but shared with S2)

P5_S4: CN LOH: 1p36.12-p32.3 (S1, S2, S3)

Sub clonal events:

P5_S1: CN LOH: 1p36.33-1p36.12

P5_S2: Gains: 1q23.2-q23.3, 3p26.3-p21.1 (minor sub clone shared with S3), 10p15.3, 18q11.2, 19q13.32-q13.43 (shared with S3)
Losses: chr18
Complex events: chr2

P5_S3: Gains: 19q13.32-q13.43 (shared with S2).

P5_S4: CN LOH: 1p36.33-p36.12 (S2, S3, and maybe S1), 19p13.3-p13.11
Losses: 1q43, 3p21.1, 3q22.1, 3q23-25.1, 3q26.32-q28

Selected A) complex alterations and B) incompatible imbalances:

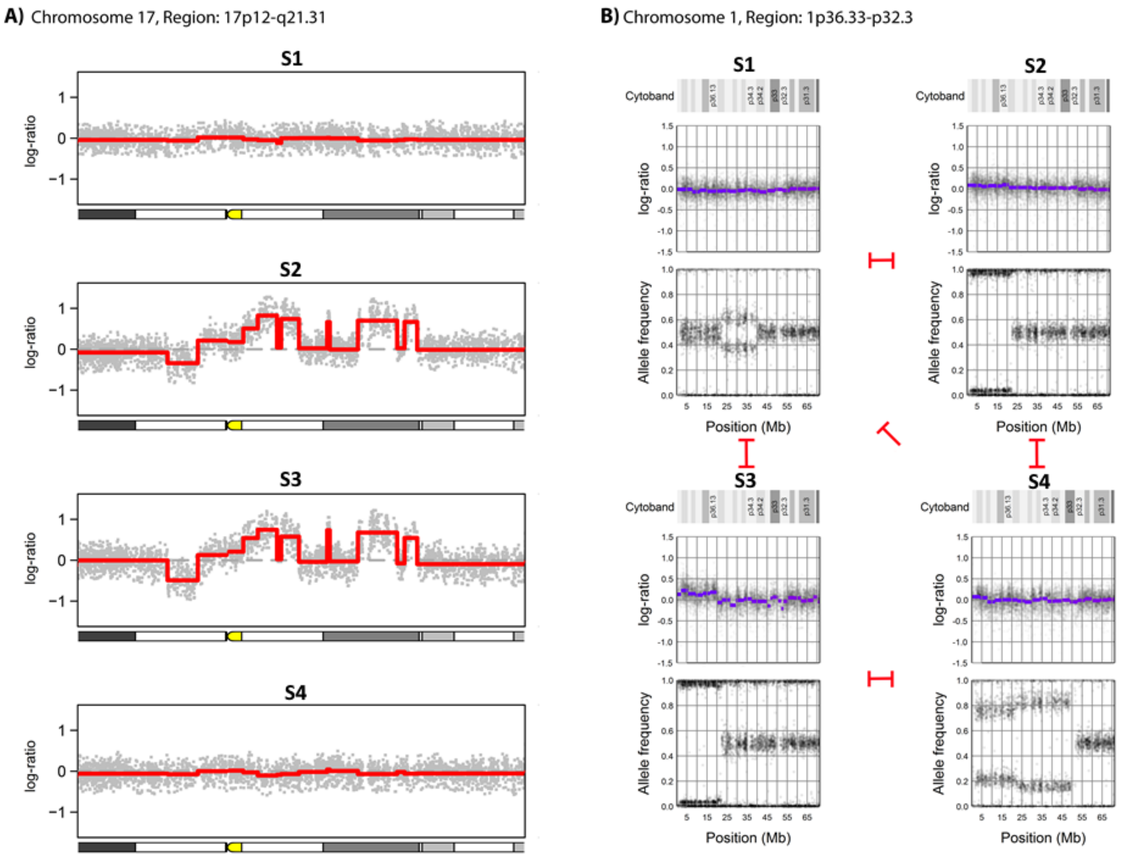

P6

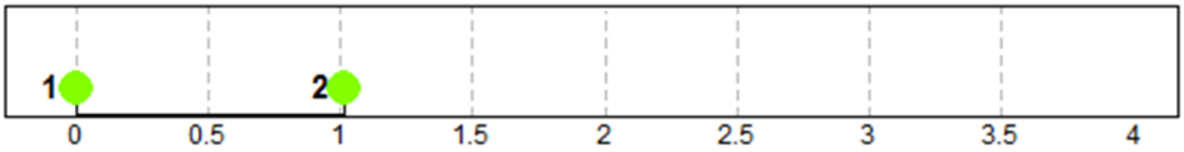

Tumor characteristics:

Sample	Pathology	Molecular subtype	Purity	Ploidy
**P6_S1**	TaG2	Uro	96%	Diploid
**P6_S2**	TaG1	Uro	84%	Diploid

Mutation data:

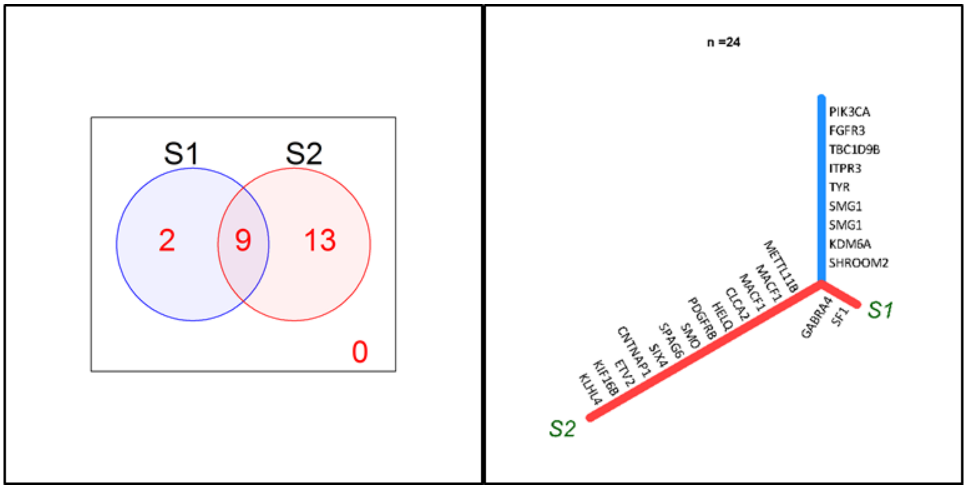

Breakpoints (window size 100K):

	P6_S1	P6_S2
**P6_S1**	**34**	6
**P6_S2**	4	**23**

Shared Imbalances:

Gains: NA

Amplifications: NA

Losses: -9

Homozygous deletions (HD): 9p21.3

Copy-neutral LOH: NA

Private alterations:

Compatible:

P6_S1: Gains: 5p

P6_S2: Gains: 17q, 19q

Incompatible events:

P6_S1: Losses: 1p31.2-p13.3, 7p14.1

P6_S2: Losses: 18q12.2

Sub clonal events:

P6_S1: Gains: 10p15.3-10p12.1, 13q21.1-13q34

Losses: chr2, 10p12.1-10q26.3, 12q24.32-12q24.33

P6_S2: NA

Selected incompatible alterations:

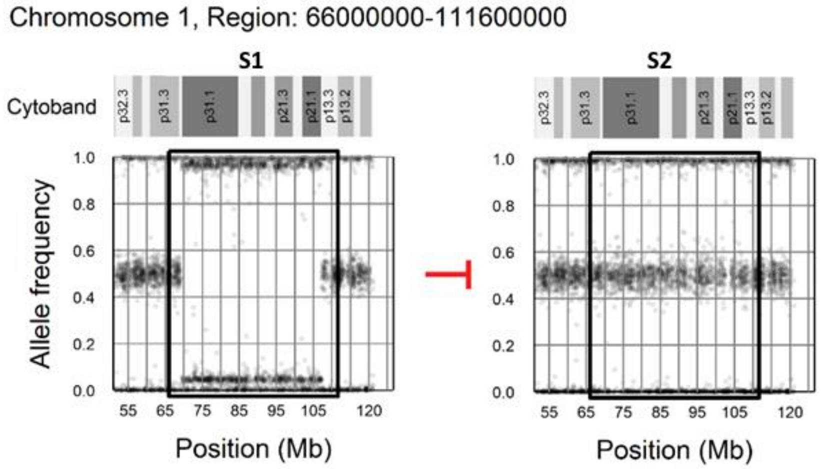

P7

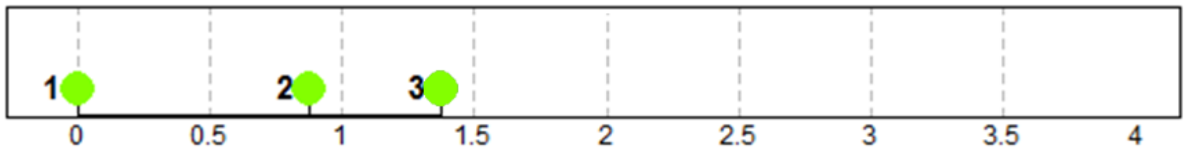

Tumor characteristics:

Sample	Pathology	Molecular subtype	Purity	Ploidy
**P7_S1**	T1G3	Uro	94%	Triploid
**P7_S2**	T1G3	Uro	85%	Triploid
**P7_S3**	T1G3	Uro	57%	Triploid

Mutation data: NA

Breakpoints (window size 100K):

	P7_S1	P7_S2	P7_S3
**P7_S1**	**202**	113	109
**P7_S2**	112	**241**	105
**P7_S3**	110	106	**209**

Shared Imbalances:

Complex events: 1q, 2q, 16p, 7q (S1&S2)

Gains: 2q23.3-q24.2, 7p22.3-p11.2 (S1&S2), 9q34.3, 17q11.2-q12 (S1&S2), 17q25.1-q25.3

Amplifications: 1p21.1-p21.1, 1q23.1-q25.2, 4p16.3, 18q12.2-q12.2 (S1&S3)

Losses: 1q44, 2q23.3, 2q32.1-q37.3, 4q21.21-q21.23, 4q24-q25, der(7q34-q36.3) (S1&S2), -9, 10q11.21-q24.1, 11q23.2-q25, -17p, 18p11.32

Homozygous deletions (HD): NA

LOH: 8p23.3-p21.3

Private alterations: Not all events can be listed due to the sample complexity.

Compatible: Not listed

Incompatible events: (major sub clones)

P7_S1: Losses: 4q32.1-q35.2 (S1 -| S2 & S1 -| S3), 7q34-q36.3 (S1 -| S3)

P7_S2: Losses: 6q16.1-q16.2 (S2 -| S1), 7q34-q36.3 (S2 -| S3)

P7_S3: Losses: chr6 (S3 -| S1 & S3 -| S2), chr15 (S3 -| S1 & S3 -| S2)

Sub clonal events: Not listed

Selected examples of A) shared, B) altered, and C) incompatible alterations:

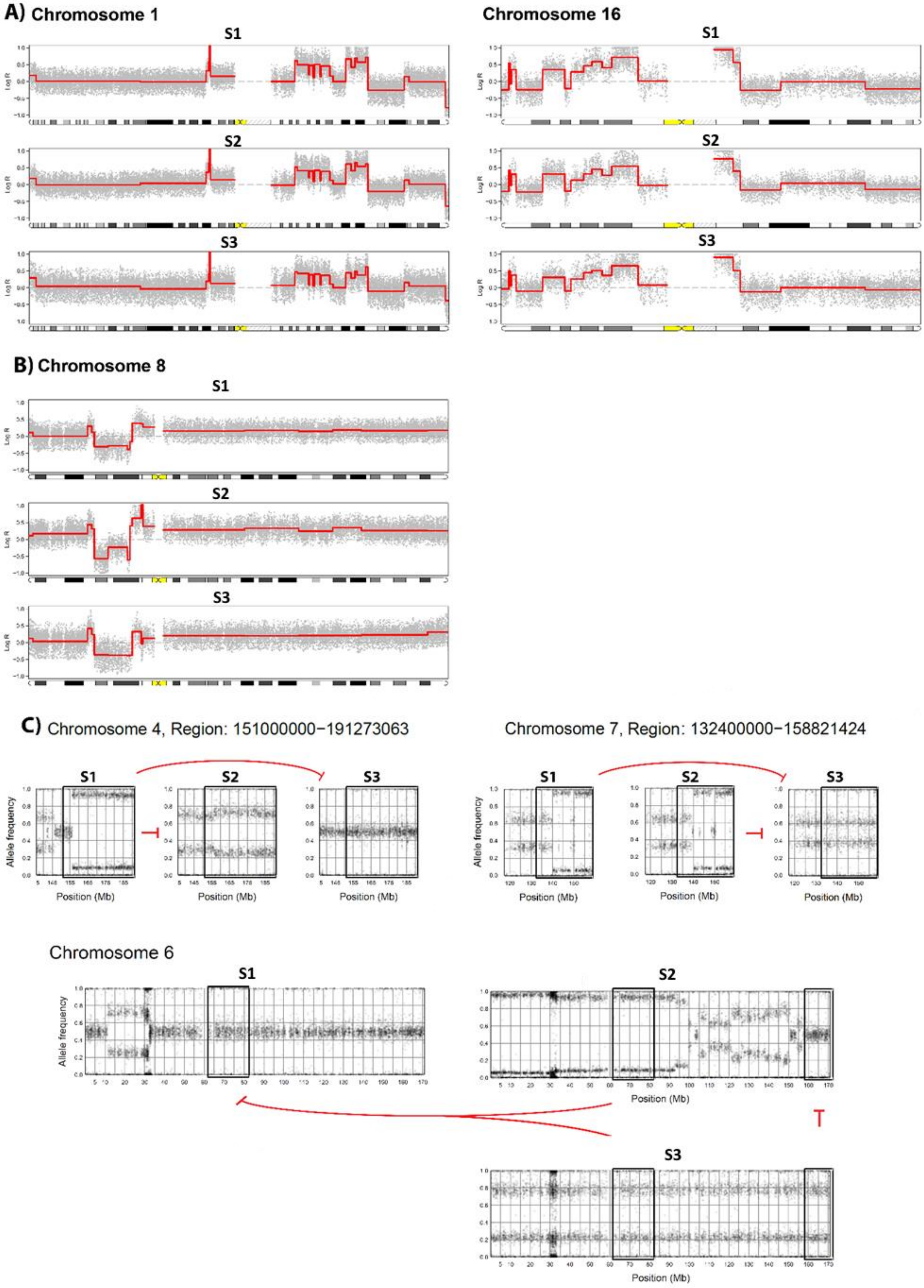

P8

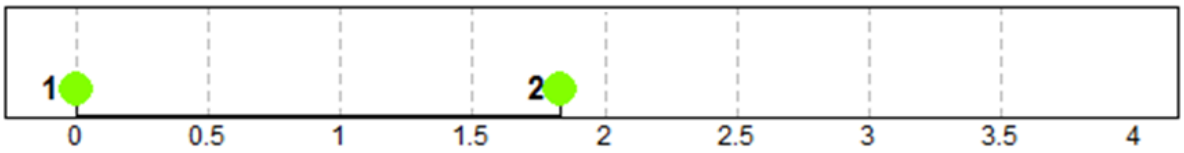

Tumor characteristics:

Sample	Pathology	Molecular subtype	Purity	Ploidy
**P8_S1**	TaG2	Uro	65%	Triploid
**P8_S2**	TaG2	Uro	91%	Triploid

Mutation data:

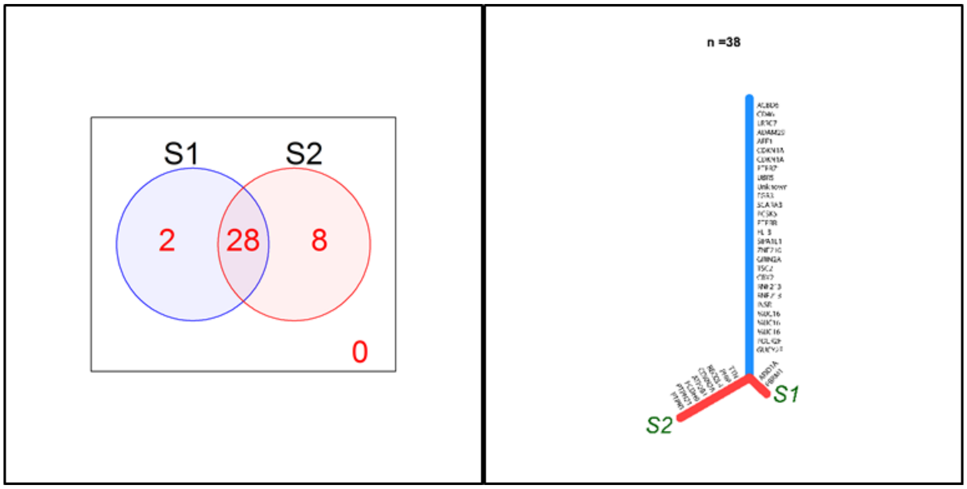

Breakpoints (window size 100K):

	P8_S1	P8_S2
**P8_S1**	**60**	23
**P8_S2**	24	**64**

Shared Imbalances:

Gains: +3, +7, +12, 12p11.22-p11.21, +16, +19, +20

Amplifications: 8q22.2-q22.3, 11q13.2-q13.3, 15q22.2, 15q22.2-q22.31

Losses: -chr2, 8q22.3, 8q23.3-q24.12, 8q24.22-q24.3, -chr9, 11p11.2-q13.2, 11q14.1-q25, 15q22.2, -chr18, -chr21

Homozygous deletions (HD): NA

Copy-neutral LOH: NA

Private alterations:

Compatible: NA

Incompatible events:

P8_S1: NA

P8_S2: Losses: 13q12.11-13q34.

CN LOH: 20p12.1

Sub clonal events:

P8_S1: NA

P8_S2: Gains: chr5

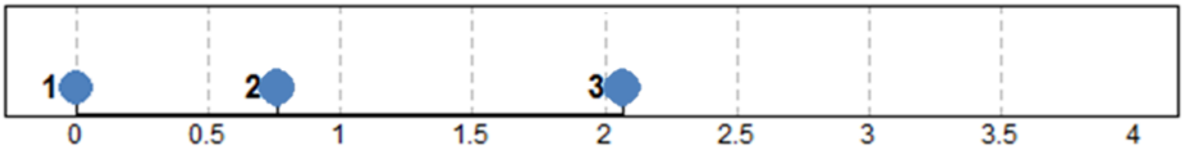

Tumor characteristics:

Sample	Pathology	Molecular subtype	IHC subtype	Purity	Ploidy
**P9_S1**	T1G3	GU[1]	GU	95%	Polyploid[3]
**P9_S2**	T2G3	Mes-like[2]	GU	<50%	Polyploid[3]
**P9_S3**	T2G3	Mes-like[2]	GU	<50%	Polyploid[3]

1) GU: Genomically Unstable. 2) Mes-like: Mesenchymal-like.
3) n is undetermined due to sample complexity.

Mutation data:

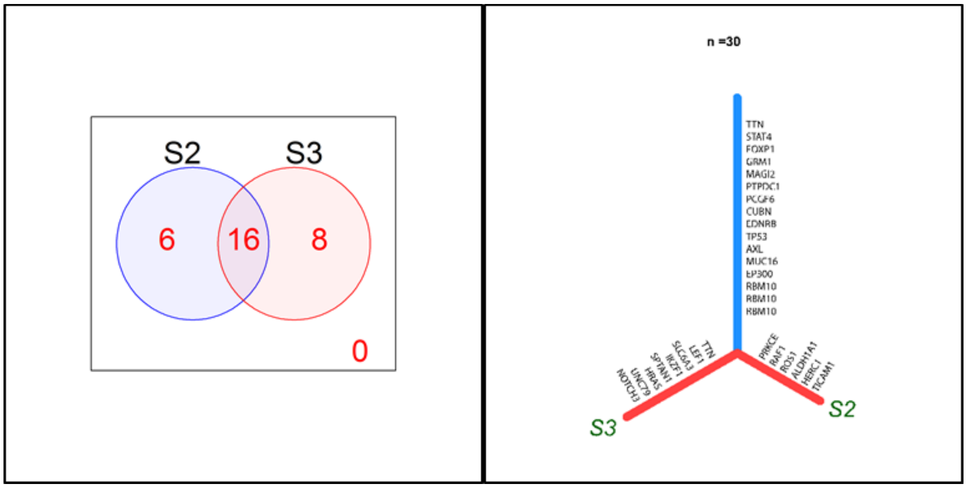

Breakpoints (window size 100K):

	P9_S1	P9_S2	P9_S3
**P9_S1**	**319**	45	55
**P9_S2**	43	**165**	46
**P9_S3**	51	47	**186**

Shared Imbalances: Not all events can be listed due to the sample complexity and low purity.

Gains: 1p36.33-p36.32, 19p13.2-p13.11 (S2 & S3)

Amplifications: 6p22.3-p22.2, 6p22.1-21.33, 16p12.1-p11.2

Losses: 10q11.23-q22.1, 10q23.1-q26.3, 13q12.2-q14.3, 4q34.1-q35.2 (S2 & S3)

Homozygous deletions (HD): Not listed

Copy-neutral LOH: Not listed

Private alterations: Not all events can be listed due to the sample complexity and low purity.

Compatible: Not listed

Incompatible events:

- **P9_S1**: Not listed
- **P9_S2**: Not listed
- **P9_S3**: Copy-neutral LOH: 11p (3 -|1, 3 -|2)

Sub clonal events: Not listed

Selected incompatible alterations:

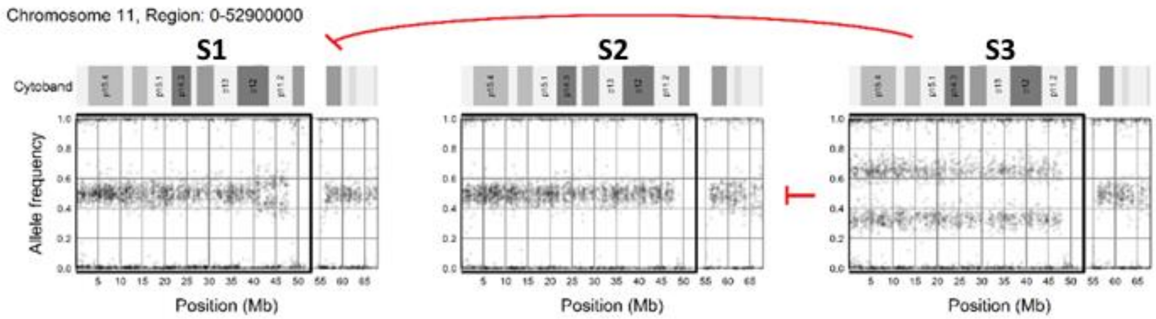

Selected private alterations:

P9: Chromosome 1

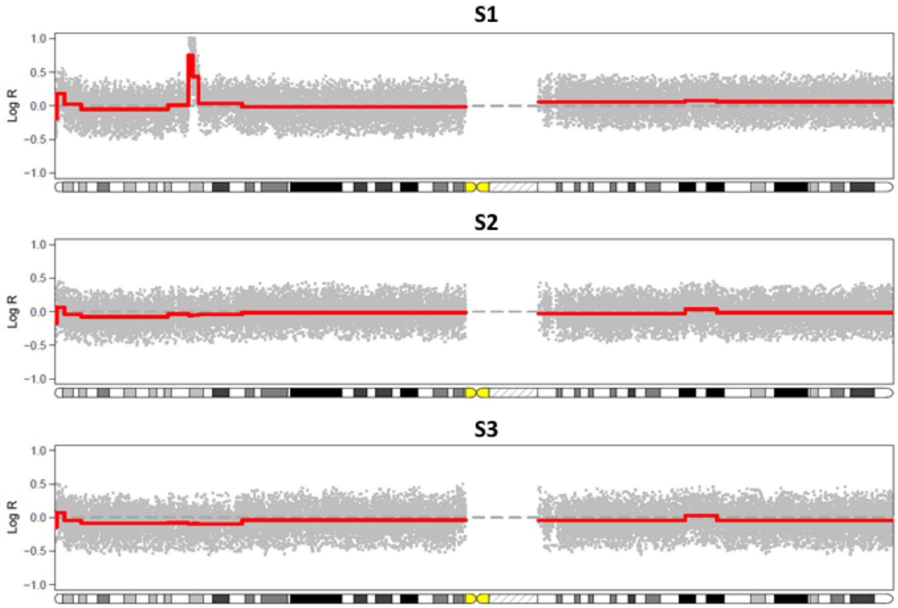

P9: chr2p

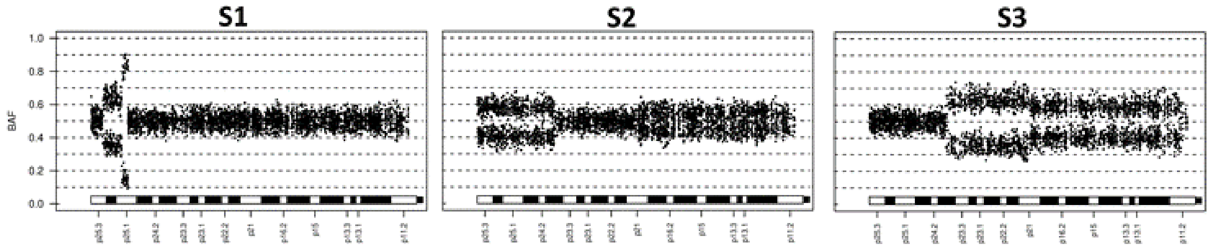

P10

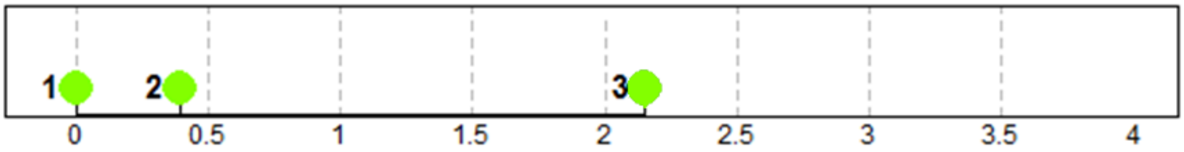

Tumor characteristics:

Sample	Pathology	Molecular subtype	Purity	Note	Ploidy
**P10_S1**	T1G2	Uro	>90%		Polyploid[1]
**P10_S2**	T1G3	Uro	86%	Resected from the same location of _1	Polyploid[1]
**P10_S3**	T2G3	Uro	35%		Polyploid[1]

1) Undetermined due to sample complexity.

Mutation data:

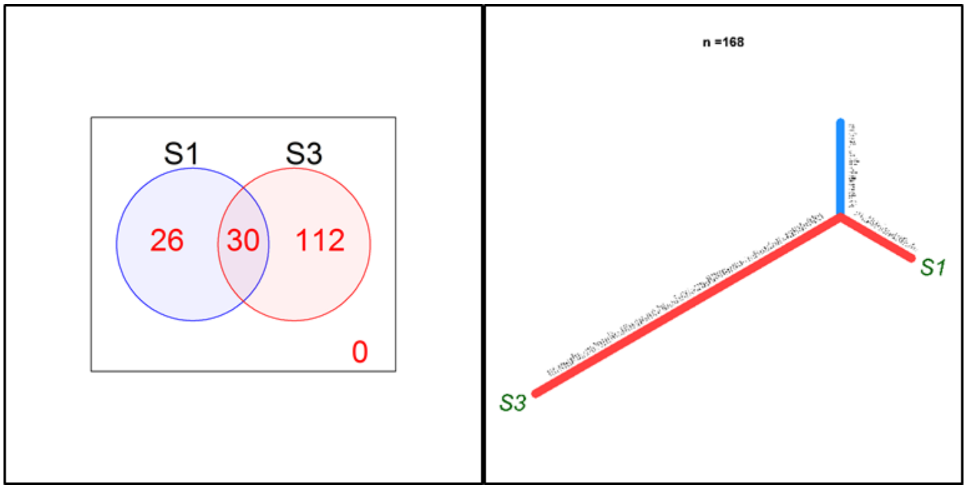

Breakpoints (window size 100K):

	P10_S1	P10_S2	P10_S3
**P10_S1**	**295**	79	50
**P10_S2**	74	**194**	53
**P10_S3**	45	52	**166**

Shared Imbalances: Not all events can be listed due to the sample complexity and subclonality.

Gains: 3q26.31-q29 (in S2&S3)
Amplifications: 10q21.3-q22.3, 20q12-q13.12,
Losses: 18q22.1-q22.2, parts of 10q (in S2&S3).
Homozygous deletions (HD): 10q25.2-q25.3 (in S2&S3)
Copy-neutral LOH: Not listed

Private alterations: Not all events can be listed due to the sample complexity and subclonality.

Compatible: Not listed

Incompatible events: No clear incompatible events

Sub clonal events: Not all events are listed

Gains: 4q13.2-q25, 7q11.21-q21.12, 8q21.3 (S1&S2), 8q24.21, 10q21.1-21.3 (S1&S2)
Losses: 1p36.31-p31.3, 1p36.21, 2q37.1, 17q21.31-q21.32
Homozygous deletions (HD): 10q25.2-q25.3 (S1)

Selected private complex alterations:

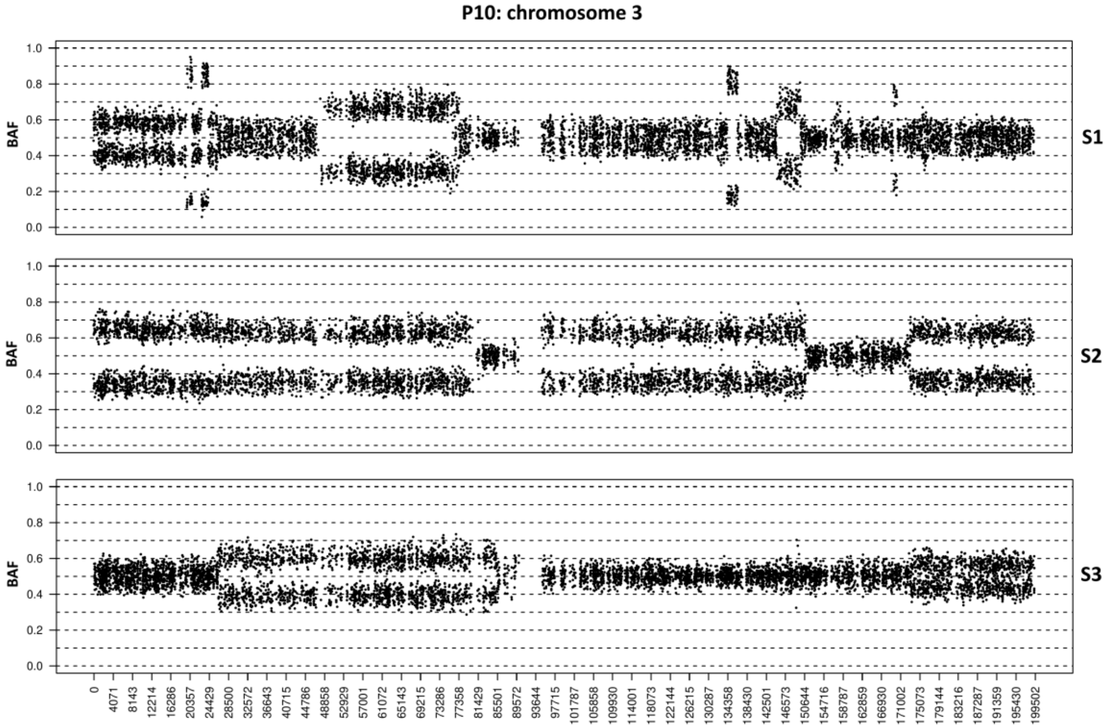

P11

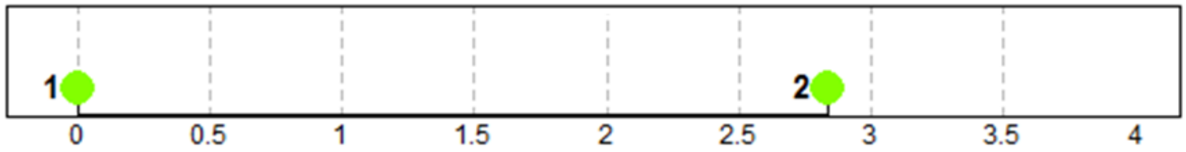

Tumor characteristics:

Sample	Pathology	Molecular subtype	Purity	Note	Ploidy
**P11_S1**	TaG2	Uro	92%		Diploid
**P11_S2**	TxGx	Uro	80%	Resected from the same location	Diploid

Mutation data: NA

Breakpoints (window size 100K):

	**P11_S1**	**P11_S2**
**P11_S1**	**20**	7
**P11_S2**	8	**18**

Shared Imbalances:

Gains: 1q, 17q24.3-q25.3
Amplifications: NA
Losses: chr9, 17p13.3-p11.2
Homozygous deletions (HD): NA
Copy-neutral LOH: 4p16.3-p13

Private alterations:

Compatible: NA

Incompatible events: NA

Sub clonal events:

P11_S1: Gains: chr16
P11_S2: NA

Selected shared alterations:

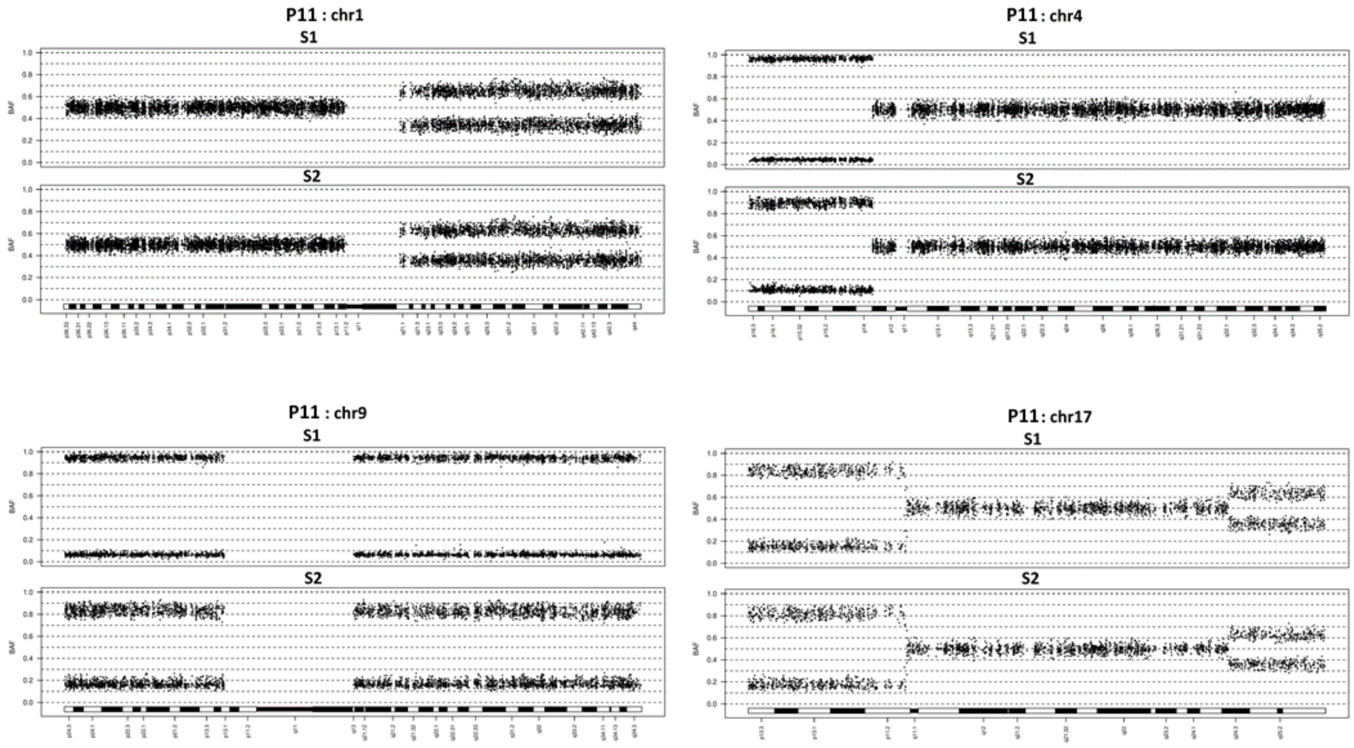

P12

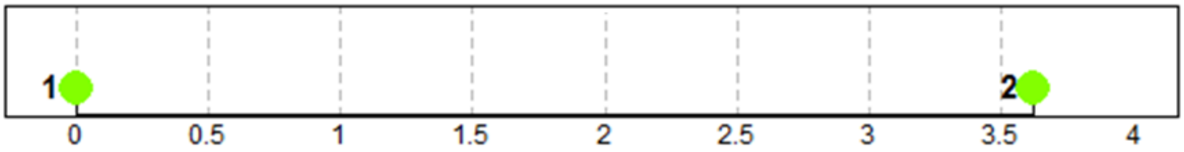

Tumor characteristics:

Sample	Pathology	Molecular subtype	Purity	Ploidy
**P12_S1**	TaG2	Uro	93%	Diploid
**P12_S2**	TaG1	Uro	63%	Diploid

Mutation data: NA

Breakpoints (window size 100K):

	P12_S1	P12_S2
**P12_S1**	**51**	17
**P12_S2**	17	**59**

Shared Imbalances:

Gains: NA

Amplifications: NA

Losses: 11q23.3-q24.1, 9p, 9q, 12p13.33-p13.32, 13q13.3-q21.32, 13q22.2-q22.3, 13q22.3-q32.1

Homozygous deletions (HD): NA

Private alterations:

Compatible:

P12_S1: Gains**:** 1q, 13q11-13q13.3, 13q21.33-13q22.2, 13q22.3, 13q32.2-13q34

P12_S2: Gains**:** 1q21.2, 1q23.1-q44

Incompatible:

P12_S1: Losses**:** 2p16.3, 4p15.32-4p15.31, 11p15.2-p14.3

HD**:** 9p23, 9p21.3, 9q22.31,9q22.32

P12_S2: Losses**:** 4q21.22, 6q16.1-q27, 8p23.3-p12, 18p11.31

HD**:** 9p21.3

Sub clonal events:

P12_S1: Losses: 1q43, 12q24.33, 16p13.3

P12_S2: Gains: 21

Selected incompatible alterations:

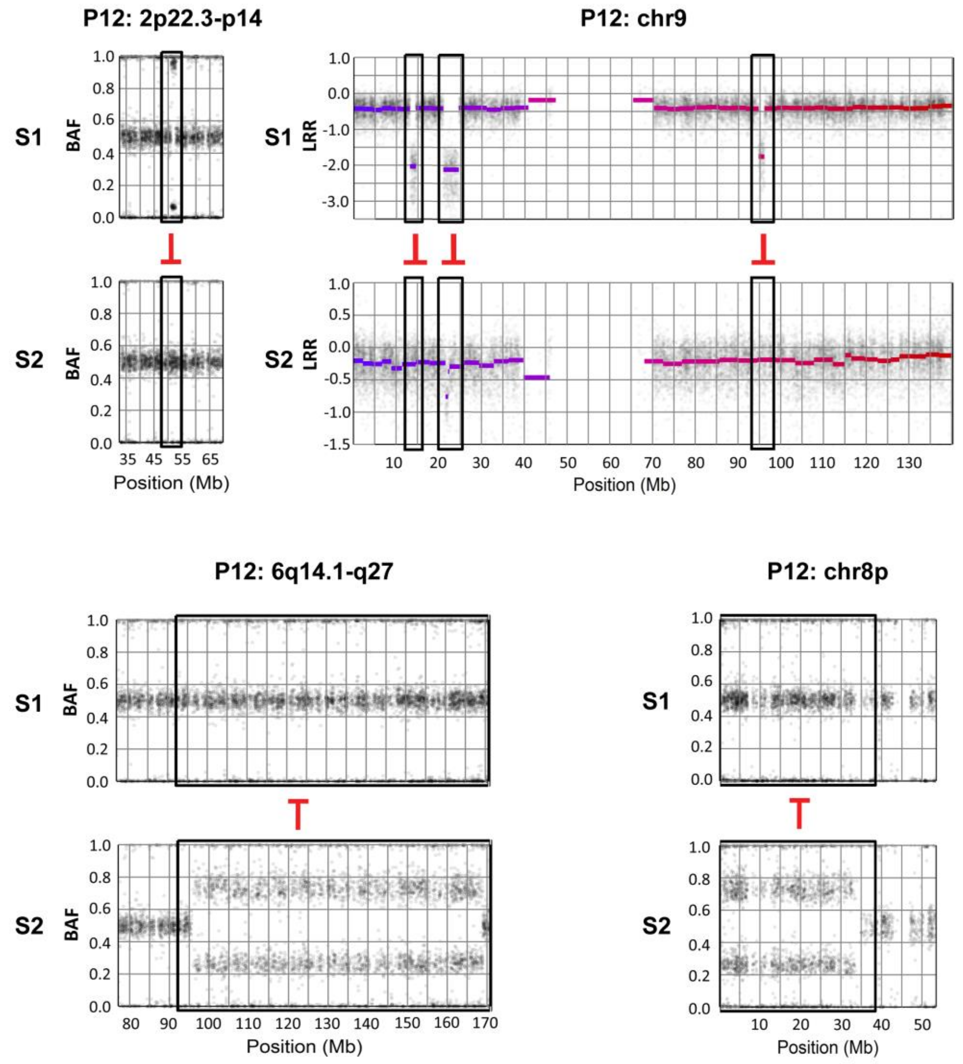

Selected reciprocal incompatible alterations:

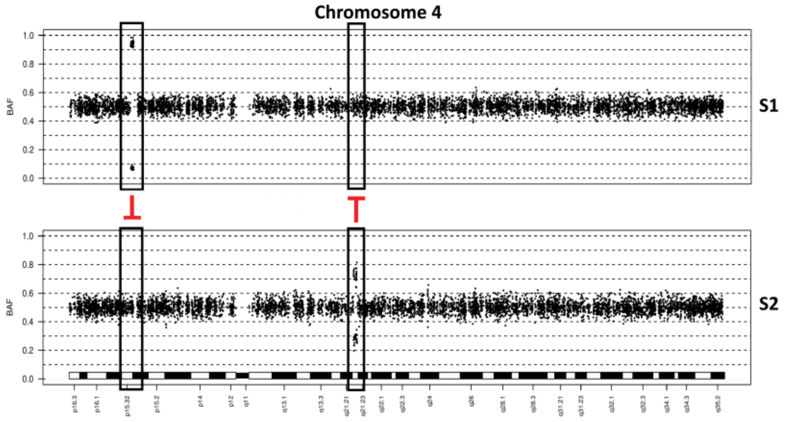

P13

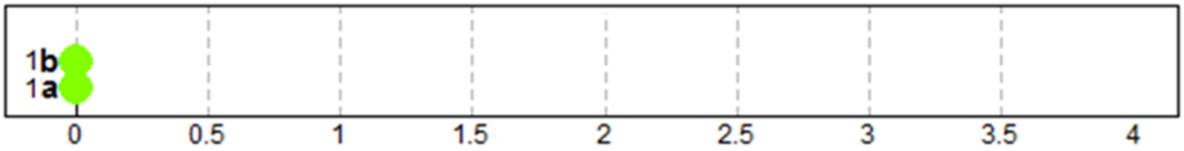

Tumor characteristics:

Sample	Pathology	Molecular subtype	Purity	Ploidy
**P13_S1a**	TaG1	Uro	32%	Diploid
**P13_S1b**	TaG2	Uro	40%	Diploid

Mutation data: NA

Breakpoints (window size 100K):

	P13_S1a	P13_S1b
**P13_S1a**	**17**	2
**P13_S1b**	2	**15**

Shared Imbalances:

Gains: NA
Amplifications: NA
Losses: NA
Homozygous deletions (HD): NA
Copy-neutral LOH: 9p21.1-p13.3

Private alterations:

Compatible: NA

Incompatible: NA

Sub clonal events: NA

Selected shared alterations:

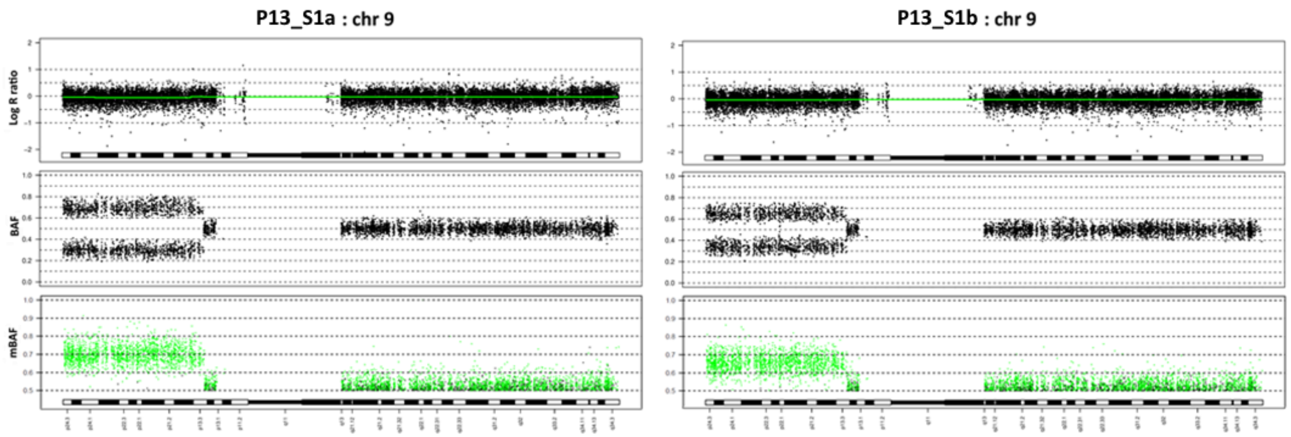

Copy-neutral LOH on chromosome 9 in both samples of case P13. The region with only green dots (SNPs) represents the allelic imbalance that occurred on the same haplotype in both samples. The region with black dots represents regions with no allelic imbalance.

P14

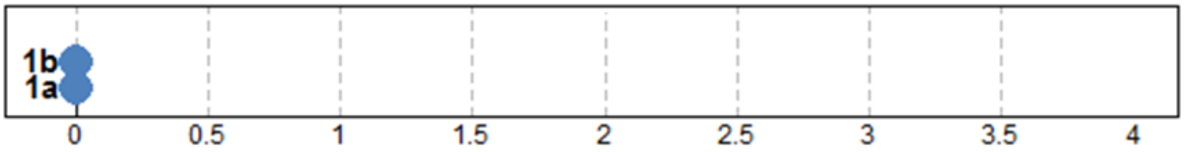

Tumor characteristics:

Sample	Pathology	Molecular subtype	Purity	Note	Ploidy
**P14_S1a**	T2G3	GU	94%		Triploid
**P14_S1b**	T2G3	GU	78%		Triploid

Mutation data: NA

Breakpoints (window size 100K):

	P14_S1a	P14_S1b
**P14_S1a**	**114**	9
**P14_S1b**	9	**199**

Shared Imbalances:

Gains: 8q12.1-q24.3

Amplifications: NA

Losses: 5q, 13q12.3-q21.2

Homozygous deletions (HD): NA

Copy-neutral LOH (3n): NA

Private alterations:

Compatible:

P14_S1a: **Gains:** 1p, 1q23.1-q23.3, 2q31.3-32.1, chr3, 4q35.1-q35.2, 5p15.33-p14.1, 5p14.1-p11, 8p11.21-8p11.1, 8q12.1, chr9, 11q23.3-q25, der(13), 15q13.3, 16p13.3-12.3, 17p13.3-13.2, 17p13.2-p13.1, 17p12, 17p11.1-q25.3, 18p11.32-q11.2, 20p13-p11.1, 20q11.21-q11.22, 20q11.23-q13.33, chr21, 22q11.21-q12-1, 22q12.1-q13.2

Losses: 2p25.1, 2q32.1-q37.3, -9p, 15q, 17p13.3, 17p13.1-p12, 17p12-p11.1

P14_S1b: **Gains:** 1q21.1-q41, 1q41, 1q41-q44, 2p25.3-p24.1, 2p, 2q11.2-12.1, chr3, 4p14-p12, 4q27-28.2, 6p22.3-p22.1, 6q14.1, 6q13.3-q21, 6q22.1-q22.31, 6q24.1-q24.3, 7p22.3-q11.22, 8q12.1-q24.3, 10p15.3-12.33, 10p12.33-p11.1, 16p11.2-p11.1, 17p11.2, 18p11.32, 18p11.32-p11.31, 18p11.31, 18p11.22, 18p11.21, 19p13.2-p13.13, 20q11.21-q12, 20q13.11-q13.13, 22q12.3-13.2

CN LOH: 8p11.21-8q12.1, 14q32.2-33-3

Incompatible events:

P14_S1a: Losses: 11q23.2-q23.3, 15q13.3-q15.1, 16p11.1, 16q12.1-q24.3, 20q11.22-q11.23, 22q13.2-q13.33

CN LOH: 7p22.3-p21.3, 8p23.3-p11.22, 18q11.2-q23

P14_S1b: Losses: parts of chr4, 8p23.3-p21.3, 10q, 18q, -22

Sub clonal events:

P14_S1a: Gains: 4q.
Losses: 4p

P14_S1b: Gains: on chr4, 16p12.3-p11.2, chr19.
CN LOH: on chr6.
Losses: 9p.

Selected private complex alterations:

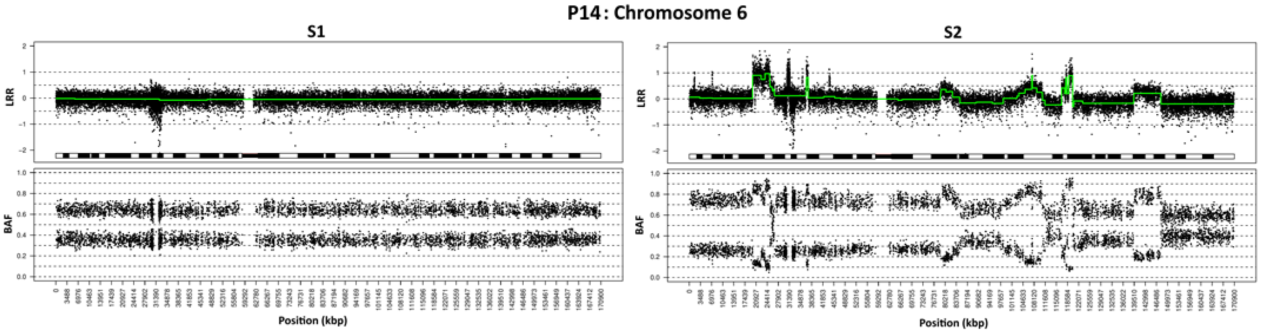

Selected incompatible alteration:

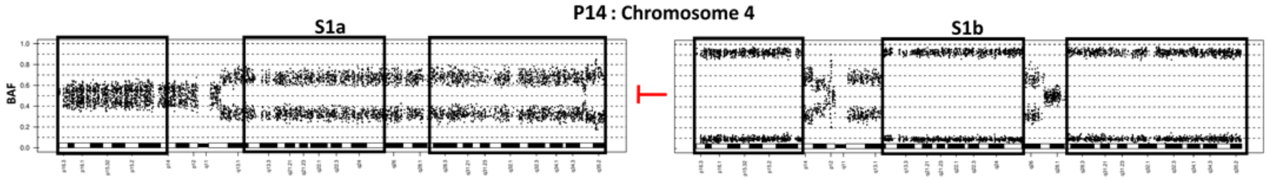

P15

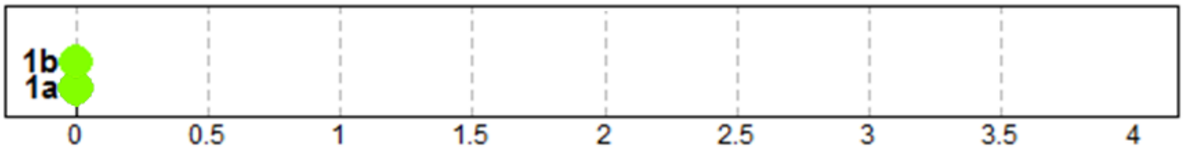

Tumor characteristics:

Sample	Pathology	Molecular subtype	Purity	Ploidy
**P15_S1a**	T1G3	Uro	>85%	Triploid
**P15_S1b**	T1G3	Uro	40%	Triploid

Mutation data: NA

Breakpoints (window size 100K):

	P15_S1a	P15_S1b
**P15_S1a**	**101**	24
**P15_S1b**	25	**90**

Shared Imbalances*:

Gains: Not listed

Amplifications: 14q11.2, 20q13.12

Losses: 8q24.11-q24.12

Homozygous deletions (HD): Not listed

Copy-neutral LOH: Not listed

Private alterations*:

Compatible:

P15_S1a: Gains: 3p25.2-p25.1

P15_S1b: Gains: 3p26.3-q11.2

Incompatible events:

P15_S1a: Copy-neutral LOH: 11q

P15_S1b: Copy-neutral LOH: 2p21-p25.3

Sub clonal events*

*) Not all events can be listed due to the complex sub clonality.

Selected private alterations:

P15 : chr3

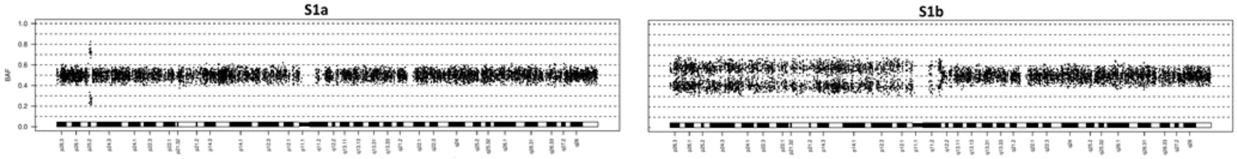

Selected incompatible alterations:

P15: chr2

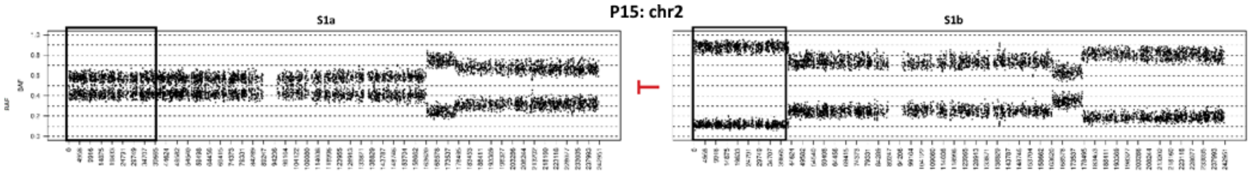

P15: chr 11

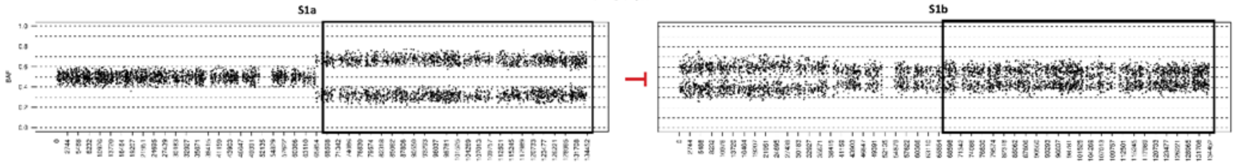

P16

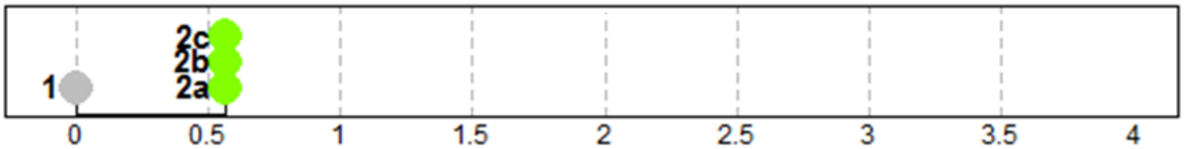

Tumor characteristics:

Sample	Pathology	Molecular subtype	Purity	Ploidy
**P16_S1**	TaG2	ND[1]	28%	Diploid
**P16_S2a**	TaG2	Uro	80%	Diploid
**P16_S2b**	TaG1	Uro	94%	Diploid
**P16_S2c**	TaG1	Uro	96%	Diploid

1) Not determined due to lack of gene expression data

Mutation data: NA

Breakpoints (window size 100K):

	P16_S1	P16_S2a	P16_S2b	P16_S2c
**P16_S1**	**19**	5	6	4
**P16_S2a**	5	**36**	10	8
**P16_S2b**	6	10	**26**	9
**P16_S2c**	4	8	9	**13**

Shared Imbalances:

Gains: chr8 (S2a, S2b & S2c)
Amplifications: NA
Losses: 14q31.1
Homozygous deletions (HD): 9p21.3
Copy-neutral LOH: 6p25.3-p21.1, chr9

Private alterations:

Compatible: NA

Incompatible events: NA

Sub clonal events:

P16_S1: Copy-neutral LOH: 6p25.3-p21.1
Losses: 14q31.1
P16_S2a: NA
P16_S2b: NA
P16_S2c: NA

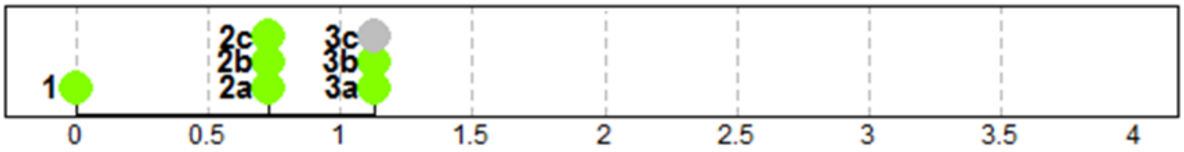

Tumor characteristics:

Sample	Pathology	Molecular subtype	Purity	Ploidy
**P17_S1**	TaG2	Uro	95%	Diploid
**P17_S2a**	TaG1	Uro	78%	Diploid
**P17_S2b**	TaG1	Uro	78%	Diploid
**P17_S2c**	TaG1	Uro	88%	Diploid
**P17_S3a**	TaG2	Uro	75%	Diploid
**P17_S3b**	TaG2	Uro	72%	Diploid
**P17_S3c**	TaG1	ND[1]	89%	Diploid

1) Not determined due to lack of gene expression data

Mutation data:

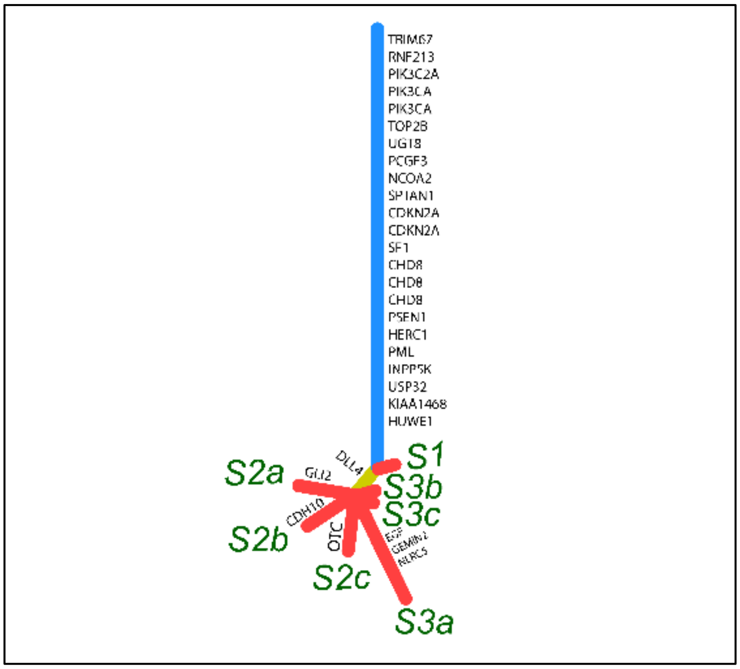

Breakpoints (window size 100K):

	0354_1	0354_2a	0354_2b	0354_2c	0354_3a	0354_3b	0354_3c
**P17_S1**	**38**	7	2	9	4	6	5
**P17_S2a**	6	**50**	23	25	33	30	26
**P17_S2b**	2	23	**49**	24	26	29	23
**P17_S2c**	9	26	24	**71**	30	31	25
**P17_S3a**	4	33	26	30	**71**	33	28
**P17_S3b**	5	30	29	32	33	**65**	29
**P17_S3c**	5	25	23	25	28	28	**59**

Shared Imbalances:
Complex event: 11q (2a-3c)
Gains: 10p15.3-p11.23 (2a-3c), 19p13.3-p13.2 (2a-3c), 19q13.11-q13.43 (2a-3c)
Amplifications: NA
Losses: 2q37.3, -9, 5q11.2-q14.3 (2a-3c), 7p22.3-p12.2 (2a-3c), 8p (2a-3c), 10q (2a-3c)
Homozygous deletions (HD): NA

Private alterations:

Compatible: NA

Incompatible events:

P17_S1: Losses: 11q14.1-q14.3 (1 main clone is incompatible with 2a-3c)
P17_S2a: Losses: 11p15.5-p11.12 (2a) -| (2b,3c)
P17_S2b: Losses: 11p15.5-p12 (2b) -| (2a,2c,3a,3b)
P17_S2c: Losses: 11p15.5-p11.12 (2c) -| (2b,3c)
P17_S3a: Losses: 11p15.5-p11.12 (3a) -| (2b,3c)
P17_S3b: Losses: 11p15.5-p11.12 (3b) -| (2b,3c)
P17_S3c: Losses: 11p15.5-p12 (3c) -| (2a,2c,3a,3b)
13q14.11-q34 (3c -| all)
17p13.3-p11.2 (3c -| all)
20p12.1 (3c -| all)

Sub clonal events:

P17_S1: Parts of chr11 complex events
P17_S2a: NA
P17_S2b: NA
P17_S2c: Parts of chr11 complex events
P17_S3a: Losses: 14q21.3-q32.33
P17_S3b: NA
P17_S3c: NA

Selected incompatible alteration:

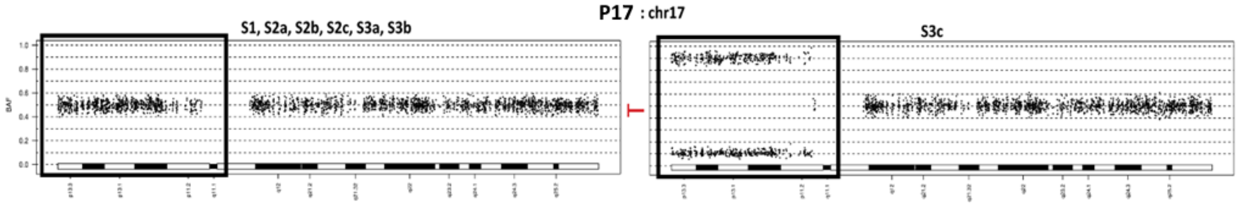

Selected complex alterations:

P17 : Chromosome 11

S1

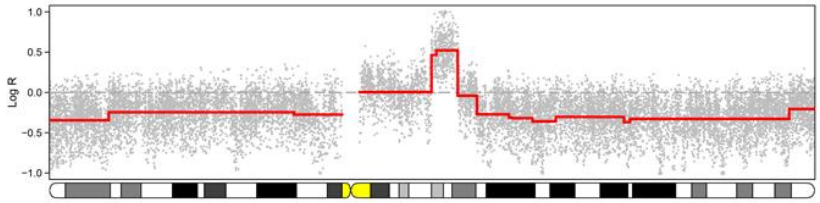

S2a

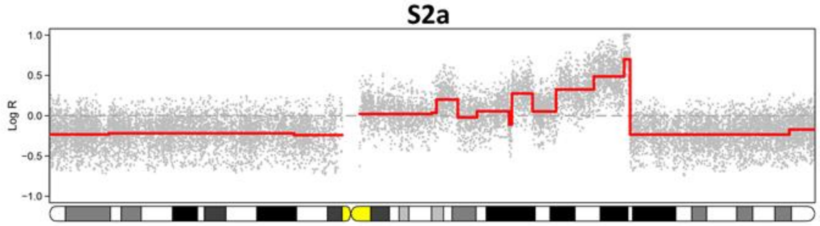

S2b

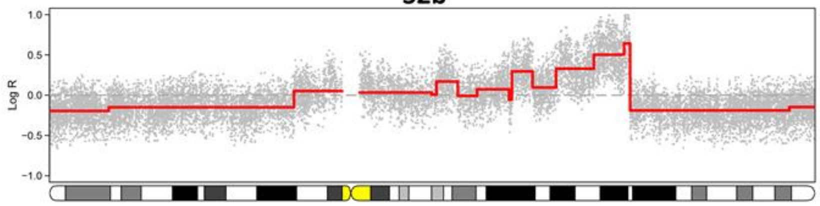

S2c

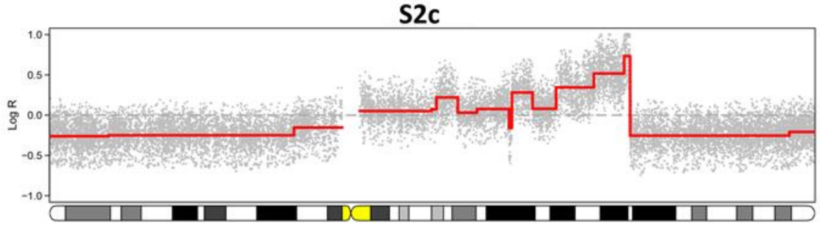

S3a

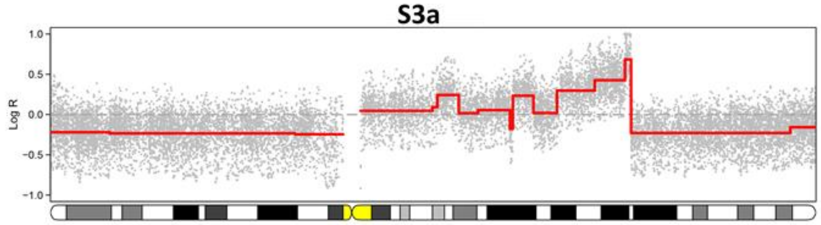

S3b

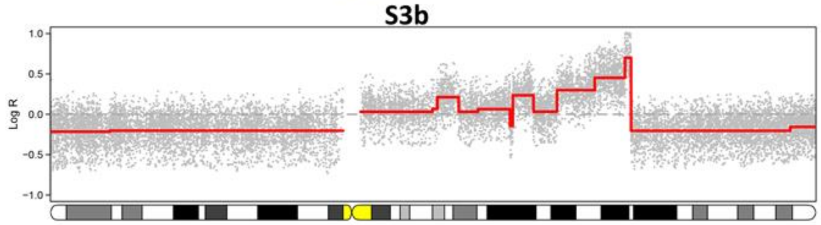

S3c

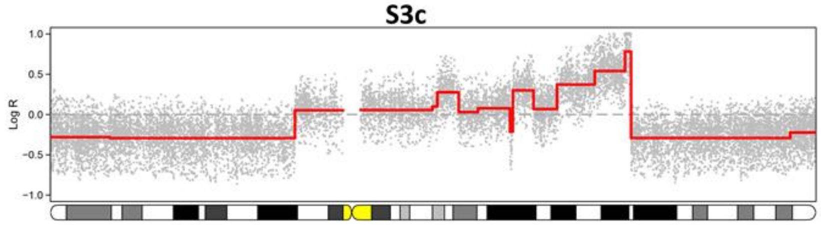

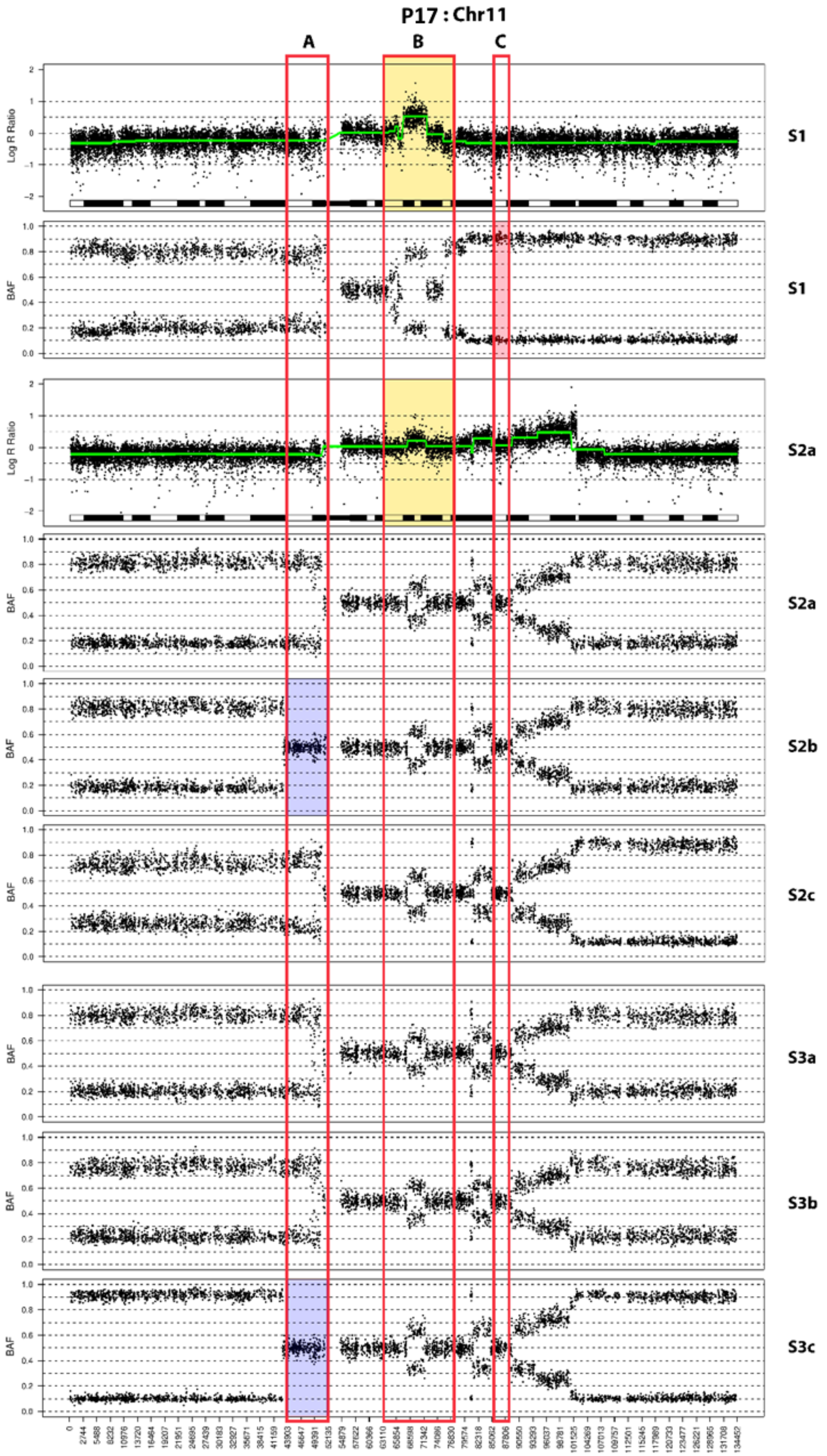

Highly rearranged chromosome 11. **A)** A deletion on 11p exists all samples except S2b and S3c, so all sample are incompatible with S2b and S3c. **B)** S1 has a gain on 11q13 (including *CCND1*) but this gain/amplification was different from the shared gain on 11q13 (also including *CCND1*) in the other six samples. **C)** The first metachronous tumor S1 showed small region of allelic imbalance in 11q14, incompatible with all the subsequent synchronous tumors.

P18

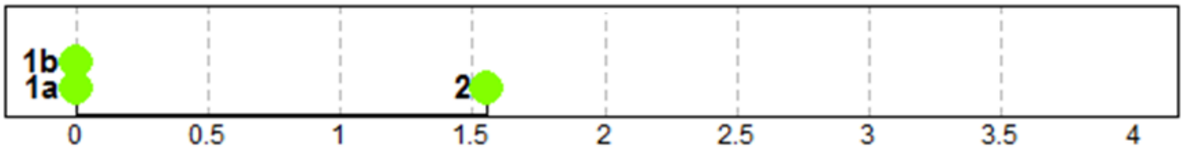

Tumor characteristics:

Sample	Pathology	Molecular subtype	Purity	Ploidy
**P18_S1a**	TaG1	Uro	92%	Diploid
**P18_S1b**	PUNLMP[1]	Uro	28%	Diploid
**P18_S2**	TaG1	Uro	22%	Diploid

1) PUNLMP: Papillary urothelial neoplasm of low malignant potential

Mutation data:

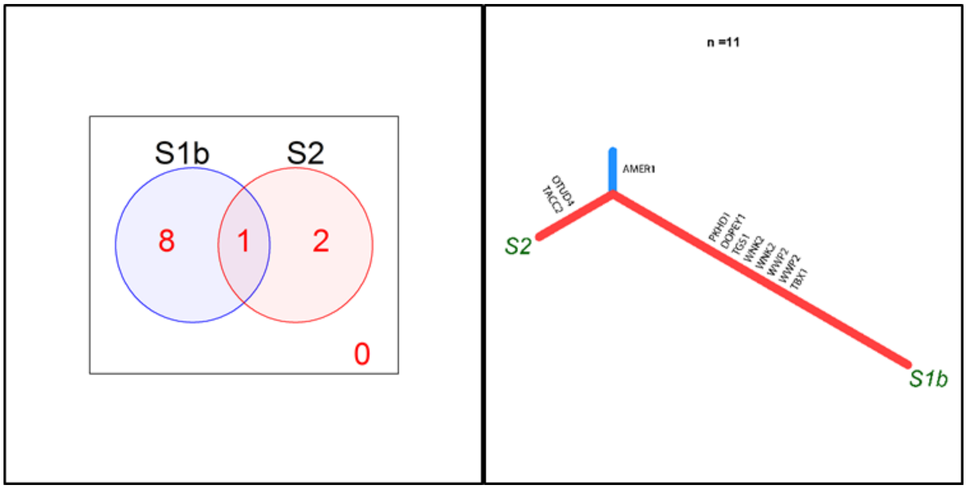

Breakpoints (window size 100K):

	P18_S1a	P18_S1b	P18_S2
**P18_S1a**	**50**	12	14
**P18_S1b**	12	**31**	10
**P18_S2**	14	9	**42**

Shared Imbalances:

Gains: 3p26.3-p22.2, 2p25.3-p22.3 (S1a & S1b), chr20 (S1a &S1b)

Amplifications: NA

Losses: 4q31.3-q35.2, 6q14.1-q22.31, 13q12.2-q14.3, 12p13.33-p12.3 (S1a &S1b)

Homozygous deletions (HD): NA

Copy-neutral LOH: NA

Private alterations:

Compatible: NA

Incompatible events:

P18_S1a: Losses: 3p14.2, 4q22.1, 7q11.22

P18_S1b: NA

P18_S2: NA

Sub clonal events:

P18_S1a: NA

P18_S1b: Losses: 4q31.3-q35.2, 12p13.33-p12.3 (S1a &S1b), 13q12.2-q14.3

P18_S2: NA

Selected incompatible alteration:

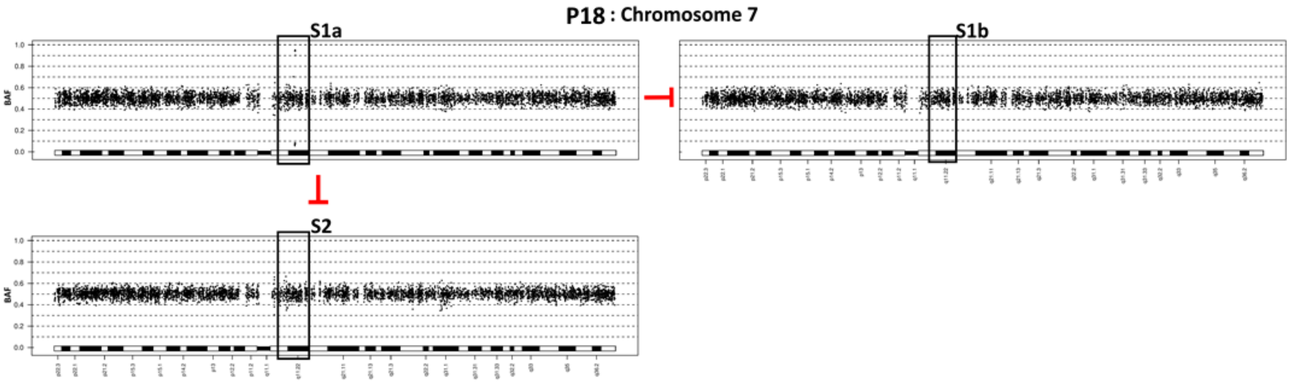

Note: All figures in supplementary file 2 were generated in R v3.6 (https://www.r-project.org/) using the packages: TAPS v2 (http://patchwork.r-forge.r-project.org/), copynumber v1.28.0 (https://bioconductor.org/), and BAFsegmentation v1.2.0, (http://baseplugins.thep.lu.se/wiki/se.lu.onk.BAFsegmentation).
